# Residual Stresses and Distortion in Material Extrusion Additive Manufacturing of Reinforced Thermoplastic Composites: A Review

**DOI:** 10.3390/polym18151796

**Published:** 2026-07-23

**Authors:** Karol Goryl, Adrián Vodilka, Marek Kočiško

**Affiliations:** Faculty of Manufacturing Technologies, Technical University of Košice, Bayerova 1, 080 01 Prešov, Slovakia; karol.goryl@tuke.sk (K.G.); adrian.vodilka@tuke.sk (A.V.)

**Keywords:** residual stress, distortion, warpage, material extrusion, fused filament fabrication, reinforced thermoplastic composites, fiber-reinforced polymers, crystallization shrinkage, process simulation, annealing

## Abstract

Material extrusion additive manufacturing, commonly implemented as fused deposition modeling (FDM) or fused filament fabrication (FFF), has evolved into a manufacturing route for reinforced thermoplastic composites, including particle-filled, short-fiber-reinforced, and continuous-fiber-reinforced systems. The process is governed by a layer-by-layer thermal cycle. Deposited roads cool rapidly, are repeatedly reheated by subsequent material deposition, and finally cool non-uniformly as part of the growing structure. This thermal history generates residual-stress that may cause warpage, build–platform detachment, delamination, dimensional error, and reduced mechanical performance. This review synthesizes residual-stress formation, measurement, modeling, parameter effects, and mitigation in material-extruded reinforced thermoplastic composites, with emphasis on short and continuous-fiber systems. Stress formation is discussed in terms of constrained thermal contraction, crystallization shrinkage, anisotropic stiffness, fiber-constrained deformation, porosity, and fiber–matrix thermal expansion mismatch. Experimental methods, including hole drilling, layer removal, curvature methods, embedded fiber Bragg gratings, digital image correlation, photoelasticity, and warpage metrology, are critically compared for anisotropic and porous printed composites. Analytical and numerical models are reviewed from layerwise shrinkage formulations to crystallization-coupled thermo-viscoelastic finite element simulations. Finally, mitigation strategies are evaluated. A central conclusion is that reinforcement can suppress visible distortion while increasing stress retained in a stiffer structure. Therefore, warpage alone is not a sufficient residual stress metric.

## 1. Introduction

Additive manufacturing (AM) builds three-dimensional objects through the progressive layer-by-layer deposition of material directly from digital models, enabling geometric complexity, mass customization, and short development cycles that are difficult to achieve with formative or subtractive processes [1,2]. Among the seven AM process categories defined in ISO/ASTM 52900 [2], material extrusion (MEX), often referred to by the trademarked term fused deposition modeling (FDM) or the generic term fused filament fabrication (FFF), is one of the most widely adopted polymer AM processes because of its relatively low equipment and feedstock cost, operational simplicity, and broad material palette [2,3,4,5]. In FFF, a thermoplastic feedstock is melted in a heated liquefier, extruded through a nozzle, and deposited as roads that solidify and bond to previously deposited material. The part is therefore built road-by-road and layer-by-layer [3,6].

The mechanical performance of neat thermoplastics, however, limits the use of FFF parts in load-bearing applications. This limitation has driven the development of composite feedstocks, in which the polymer matrix is reinforced with particles and nanofillers [7,8], short carbon or glass fibers [9,10,11] or continuous fibers embedded during deposition [12,13,14]. Short carbon-fiber feedstocks were also a key enabler of large-format material extrusion. Love et al. demonstrated that carbon fibers increase stiffness and thermal conductivity and reduce distortion sufficiently to allow printing outside heated chambers, described as printing “out of the oven” [15]. This development opened the way to Big Area Additive Manufacturing (BAAM) of meter-scale tooling and vehicle structures [16,17]. The literature on printed composites has grown accordingly and has been reviewed from the perspectives of materials and properties [18,19,20], continuous-fiber processing [21,22,23], short-fiber meta-analysis [24], and process modeling [25].

In this review, reinforced thermoplastic composites are understood as material-extrusion feedstocks in which a thermoplastic matrix is modified by short fibers, continuous-fibers, particles, or nanoscale fillers. The main emphasis is placed on short-fiber and continuous-fiber-reinforced systems. These systems introduce strong anisotropy in stiffness, thermal expansion, shrinkage, and interlayer bonding, which makes their residual-stress state fundamentally different from that of neat polymers. Particle and nanofilled systems are included where they provide relevant insight into shrinkage reduction, crystallization control, dimensional stability, or the distinction between residual stress and visible distortion.

Regardless of feedstock, the defining physical feature of FFF is its severe and highly localized thermal cycling. Each deposited road cools from the extrusion temperature toward the ambient or chamber temperature within seconds, is repeatedly reheated by neighboring and subsequent roads, and finally cools to room temperature together with the entire part [26,27,28]. Because the material is constrained by the previously solidified substrate, the thermal contraction associated with this history cannot occur freely. The constrained shrinkage is therefore stored partly as residual stress [29,30,31]. In semi-crystalline matrices, crystallization shrinkage adds a second, material-specific contribution [32,33,34]. The practical consequences are well documented and include warpage and edge curling [35,36], debonding from the build platform [37], interlayer delamination and cracking in high-temperature polymers such as PEEK [38,39], dimensional inaccuracy [40,41], and reductions in strength and fatigue life [42,43]. In fiber-reinforced feedstocks, the problem is further complicated by strongly anisotropic stiffness and thermal expansion, fiber-constrained shrinkage, crystallization of semi-crystalline matrices, and elevated porosity [31,34,44]. The same phenomenon can also be exploited deliberately. Programmed residual stresses can drive the shape-shifting of 4D printed structures [45,46,47].

Residual stresses in conventionally processed thermoplastic composites have been extensively discussed in relation to cooling, crystallization, fiber–matrix thermal expansion mismatch, and laminate-scale deformation. In material extrusion additive manufacturing, however, the corresponding knowledge remains fragmented. Relevant findings are distributed across studies of neat polymers, particle-filled systems, short-fiber feedstocks, continuous-fiber deposition, large-format extrusion, warpage experiments, and process simulations. Existing reviews have addressed material systems, mechanical properties, continuous-fiber printing, short-fiber reinforcement, and numerical process modeling, but residual stress has rarely been treated as the central organizing topic. A dedicated synthesis is therefore needed to connect formation mechanisms, measurement methods, modeling approaches, process parameter effects, material-specific behavior, and mitigation strategies for reinforced thermoplastic composites produced by material extrusion.

Recent publications from 2025 further justify an updated synthesis. New work has expanded the discussion of polymer composite FFF, short-fiber reinforcement, in situ deflection-based residual-stress assessment, thermo-mechanical process simulation, numerical modeling trends, rapid photoelastic stress evaluation, and post-process annealing of printed thermoplastics [18,24,48,49,50,51,52,53]. These studies show that the field is moving from qualitative warpage reduction toward experimentally supported stress assessment, validated simulation workflows, and process-level stress management. However, their findings remain distributed across measurement, modeling, material development, and post-processing studies, which reinforces the need for a review organized specifically around residual stress and distortion.

The objective of this review is to consolidate current understanding of residual stresses and distortion in material-extruded reinforced thermoplastic composites. First, the process-specific thermal and structural features governing stress formation are summarized. The mechanisms of constrained thermal contraction, crystallization shrinkage, reinforcement-induced anisotropy, fiber–matrix thermal expansion mismatch, and porosity are then analyzed. Experimental methods for residual-stress and distortion assessment are critically compared, followed by analytical, numerical, and data-driven modeling approaches. The effects of material selection, reinforcement type, process parameters, geometry, and toolpath strategy are synthesized. Finally, mitigation strategies and unresolved research challenges are identified, with particular attention to the distinction between reduced warpage and reduced residual stress.

The literature was identified through searches of Web of Science, Scopus, ScienceDirect, and publisher databases using combinations of the terms “residual-stress”, “thermal stress”, “warpage”, and “distortion” with “fused deposition modeling”, “fused filament fabrication”, “material extrusion”, and composite-related terms such as “fiber-reinforced”, “carbon fiber”, and “nanocomposite”. Emphasis was placed on the period 2013–2026, while earlier seminal works were retained where necessary. Studies were included when they provided direct or interpretable evidence relevant to residual-stress formation, measurement, prediction, or mitigation in material-extruded polymers and reinforced thermoplastic composites, including thermal history, crystallinity, porosity, interlayer bonding, dimensional distortion, process-induced strain, or post-process stress relaxation. Studies were excluded when they addressed only general mechanical, tribological, or functional performance without residual-stress-relevant information, or when they focused on non-material-extrusion additive manufacturing processes, except where measurement or modeling principles were transferable. A total of 170 sources were included after screening, and their bibliographic details were verified against publisher records and Crossref. This criterion was used to avoid conflating general mechanical property optimization with residual stress evidence.

The remainder of this review is organized as follows. Section 2 summarizes the FFF process and composite feedstock systems to the extent needed for the residual-stress discussion. Section 3 analyzes the formation mechanisms of residual stress and the specific contributions of reinforcement. Section 4 reviews experimental measurement and characterization methods and their applicability to printed composites. Section 5 covers analytical and numerical modeling, including crystallization-coupled and fiber-orientation-aware simulations. Section 6 synthesizes the effects of process parameters and material composition. Section 7 evaluates mitigation and control strategies. Section 8 identifies open challenges and future research directions.

## 2. Material Extrusion Process and Reinforced Thermoplastic Feedstocks

### 2.1. Process Principle, Bond Formation, and Thermal Window

In FFF, a solid thermoplastic filament is driven by counter-rotating pinch rollers into a heated liquefier, where it is melted and extruded through a nozzle of typically 0.25 to 1.0 mm diameter. The deposition head moves in the build plane while the platform or the print head indexes vertically between layers [3,6]. The deposited road usually has an elliptical or oblong cross-section. Its final dimensions are governed by nozzle diameter, layer height, extrusion multiplier, material flow rate, and the local thermal state of the previously deposited material. Adjacent roads and successive layers must coalesce to form a mechanically coherent solid.

Bond formation between roads is a thermally driven sintering and healing process. Initial contact and neck growth are governed by surface tension and melt viscosity [54,55]. The development of full interfacial strength requires interdiffusion and reptation of polymer chains across the interface, as described by crack-healing theory [56] and its FFF-specific extensions that account for non-isothermal healing, flow-induced disentanglement, and chain alignment [57,58,59,60]. Healing is effective only while the interface temperature remains above the glass transition temperature for amorphous polymers or within the relevant melting and crystallization window for semi-crystalline polymers. The short duration of this thermal window explains the partial bonding and interlayer weakness commonly observed in FFF parts [61,62,63,64].

**Figure 1:**
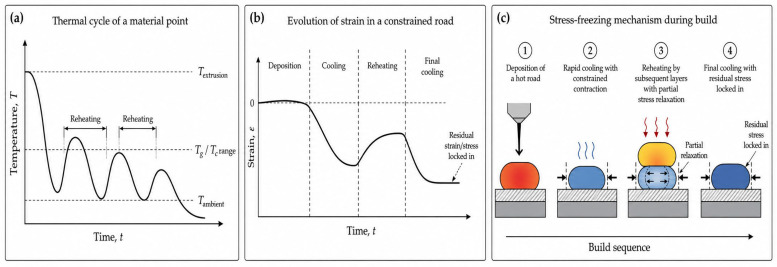
Schematic representation of residual-stress development during material extrusion. (**a**) Thermal cycle of a material point during deposition, cooling, reheating by subsequent layers, and final cooling. (**b**) Evolution of strain in a constrained deposited road, showing partial relaxation during reheating and residual strain locking during final cooling. (**c**) Stress-freezing mechanism during the build sequence, including rapid constrained cooling, reheating-induced partial relaxation, and final residual-stress retention.

The same thermal window also controls residual-stress generation. Infrared thermography and embedded thermocouple measurements show that deposited roads can cool through the glass transition within seconds. Subsequent passes generate a sequence of reheating peaks with progressively decreasing intensity [26,27,28,65]. Each road therefore solidifies against material that is already partially or fully rigid. Local reheating may relax part of the previously formed stress, but subsequent cooling regenerates stress under constraint. The resulting thermal and mechanical history is position dependent and distinguishes material extrusion from bulk polymer processes such as injection molding [30,31]. The thermal cycle and stress-freezing sequence are summarized in Figure 1.

### 2.2. Development and Recent Innovations in Material Extrusion Processes

The development of material extrusion has progressed from conventional filament-based FFF toward larger, more highly controlled, and more material-specific extrusion platforms. In conventional desktop and industrial FFF, the process is governed mainly by filament melting, pressure-driven flow through a liquefier, road deposition, inter-road coalescence, and rapid non-isothermal bonding [3,6,61,62,63,64]. This process remains the reference configuration for residual-stress analysis because the deposited roads cool rapidly, are reheated by subsequent deposition, and accumulate stress under the constraint imposed by neighboring roads, lower layers, and the build platform [26,27,28,30,31].

A major development was the transition from small-format filament extrusion to large-format material extrusion, including Big Area Additive Manufacturing (BAAM). Large-format extrusion commonly uses pellet- or high-throughput feedstock systems and short-carbon-fiber-reinforced thermoplastics to increase stiffness, thermal conductivity, and dimensional stability. Carbon-fiber-filled feedstocks enabled low-distortion printing of large components and tooling structures, but they also introduced stronger anisotropy, larger bead dimensions, slower cooling, and stronger dependence on process-scale thermal management [15,16,17,66,67]. Consequently, residual stress in large-format extrusion is not governed only by local road cooling, but also by part-scale temperature gradients, layer time, bead geometry, and anisotropic thermo-mechanical properties.

A second development is continuous-fiber material extrusion, in which carbon, glass, aramid, or other continuous reinforcements are deposited together with the thermoplastic matrix. This can be achieved through in-nozzle impregnation, co-extrusion, or deposition of pre-impregnated filaments [12,13,14,21,22]. Compared with short-fiber systems, continuous-fiber extrusion introduces much stronger path dependence because the deposited fiber trajectory defines the local stiffness, thermal contraction constraint, interfacial bonding condition, and residual strain field. Therefore, toolpath design, fiber tension, curvature, impregnation quality, and fiber–matrix bonding become process variables directly linked to residual-stress formation.

More recent process innovations have focused on high-temperature polymers and assisted thermal or thermo-mechanical control. Heated chambers and high-temperature extrusion platforms are essential for PEEK, PEKK, PAEK, and related semi-crystalline matrices because residual stress is coupled not only to thermal contraction but also to crystallization shrinkage and interlayer bonding [32,38,39,68]. Local preheating by infrared or laser sources, auxiliary heating near the deposition zone, and controlled layer-time strategies reduce thermal gradients and extend the bonding window [69,70,71]. Vacuum-assisted printing and preheating combined with mechanical compaction further modify the thermal boundary conditions, porosity, crystallization, and interlayer contact state [72,73,74]. These process developments are therefore relevant not merely as hardware improvements, but as residual-stress control strategies because they alter the temperature field, relaxation time, bonding state, crystallinity, and mechanical constraint during deposition.

Overall, the evolution of material extrusion can be interpreted as a progression from simple road-by-road deposition toward process architectures that actively control heat transfer, reinforcement placement, interlayer bonding, and part-scale constraint. This development is particularly important for reinforced thermoplastic composites, where the residual-stress state depends simultaneously on deposition sequence, thermal history, reinforcement architecture, matrix crystallization, porosity, and fiber-orientation-dependent anisotropy.

### 2.3. Reinforced Thermoplastic Feedstock Systems

Reinforced thermoplastic feedstocks for material extrusion can be classified into three principal families according to reinforcement type and length scale. The first family consists of particle and nanofilled filaments. These materials incorporate carbon nanotubes, graphene, mineral fillers, glass spheres, metal powders, wood particles, or other dispersed phases, typically to improve stiffness, electrical or thermal conductivity, crystallization behavior, dimensional stability, or functional response [7,8,75,76]. Although these materials are not fiber-reinforced in the strict sense, they are relevant to residual-stress analysis because they modify the effective coefficient of thermal expansion, the cooling response, the crystallization kinetics, and the magnitude of shrinkage-induced distortion.

The second family consists of short-fiber-reinforced filaments, most commonly containing chopped carbon or glass fibers. Typical commercial and research-grade feedstocks contain approximately 10 to 30 wt.% fiber, although higher loadings are used in large-format extrusion. During compounding, filament production, liquefier flow, and deposition, fibers undergo attrition and tend to align with the melt flow and print-path directions [9,10,24]. This orientation raises the longitudinal modulus substantially, often by a factor of two or more, but it also introduces directional stiffness and thermal expansion. As a result, short-fiber composites do not shrink isotropically. The longitudinal direction is constrained by the aligned fibers, whereas the transverse and through-thickness directions remain more matrix-dominated.

The third family includes continuous-fiber material extrusion systems. In these processes, carbon, glass, aramid, or other continuous reinforcements are deposited together with a thermoplastic matrix by in-nozzle impregnation [12,13], dual-nozzle deposition of pre-impregnated filaments [14,77,78], or co-extrusion. Continuous fibers provide the highest structural performance among material-extruded composites, but they also introduce the strongest path dependence. Local stiffness, thermal contraction, residual strain, void formation, interfacial bonding, and fiber tension depend directly on the deposited fiber trajectory, curvature, lay-up strategy, and impregnation quality [21,22,79,80]. For this reason, continuous-fiber systems require particular attention in residual-stress analysis, even though quantitative stress data for such parts remain scarce.

From the residual-stress perspective, reinforced thermoplastic feedstocks can also be classified according to their technological maturity and reinforcement architecture into traditional and advanced material systems. Traditional systems include particle-filled, nanofilled, and short-fiber-reinforced thermoplastics processed using conventional filament extrusion and standard FFF hardware. These materials mainly modify shrinkage, stiffness, thermal conductivity, crystallization behavior, and porosity, while the reinforcement remains discontinuous and statistically distributed within the deposited roads [7,8,9,10,11,24]. Advanced systems include continuous-fiber-reinforced thermoplastics, high-temperature semi-crystalline matrices such as PEEK and PAEK composites, large-format carbon-fiber-reinforced feedstocks, and multifunctional or process-tailored composites. These systems introduce stronger path dependence, higher anisotropy, more complex interfacial bonding, and stronger coupling between thermal history, fiber orientation, crystallinity, and residual stress [12,13,14,15,16,17,21,22,68]. The resulting classification of traditional and advanced reinforced thermoplastic systems is summarized in Table 1.

The principal application classes of reinforced thermoplastic material extrusion and their associated residual-stress and distortion challenges are summarized in Table 2. The intended application determines not only the required mechanical or functional performance, but also the relevant scale of thermal gradients, mechanical constraint, dimensional accuracy, and stress release.

Table 2 shows that the relevant residual-stress mechanisms and assessment requirements differ substantially among application classes. Large-format components are governed predominantly by part-scale thermal gradients, bead-level anisotropy, and platform-release distortion, whereas continuous-fiber structures introduce strong fiber-path-dependent constraint. High-temperature systems are additionally affected by crystallization shrinkage and narrow thermal-processing windows. In functional and 4D-printed structures, process-induced strain may be either an undesirable source of dimensional instability or a deliberately programmed mechanism of shape change.

This classification shows that the transition from traditional to advanced reinforced thermoplastic composites is not only a change in reinforcement content, but also a change in the dominant residual-stress mechanisms. In traditional particle- and short-fiber-filled systems, the primary effects are shrinkage reduction, stiffness increase, and porosity modification. In advanced continuous-fiber, high-temperature, and large-format systems, residual stress becomes more strongly governed by fiber path, local anisotropy, crystallization kinetics, thermal-management strategy, and deposition-scale constraint. Therefore, classification by reinforcement architecture is necessary for interpreting whether reduced distortion represents true stress reduction or only masking of stress by increased stiffness.

### 2.4. Matrix Systems: Amorphous Versus Semi-Crystalline

The matrix polymer determines both the magnitude of shrinkage and the temperature range in which stresses become locked into the printed part. Amorphous matrices such as ABS, PC, PETG, ASA, and PEI contract progressively below the glass transition temperature, Tg, and accumulate stress primarily through constrained thermal contraction [30,88]. In these systems, the key variables are the temperature interval between stress-freezing and room temperature, the coefficient of thermal expansion, the cooling rate, and the time available for viscoelastic relaxation.

Semi-crystalline matrices such as PLA, PA, PP, PEEK, and other PAEKs introduce an additional mechanism. Besides thermal contraction, they undergo crystallization shrinkage, the magnitude and timing of which depend on cooling rate, thermal history, chamber temperature, and nucleation behavior [32,89,90]. PEEK is a prominent example. Its high processing temperature and rapid crystallization make printed parts particularly prone to warpage, delamination, and cracking unless chamber and bed temperatures are tightly controlled [38,39,91,92]. Slow-crystallizing PAEK grades have therefore been developed to widen the bonding window and reduce process-induced stress, reaching a substantially higher fraction of injection-molded strength than standard PEEK [68]. Polypropylene illustrates the same problem at the commodity level. Its high crystallization shrinkage causes severe warpage and has motivated dedicated filler, adhesion, and thermal management strategies [37,81,93].

The matrix-dependent residual-stress mechanisms, characteristic manifestations, and corresponding control priorities are summarized in Table 3.

As summarized in Table 3, the distinction between amorphous and semi-crystalline matrices is central to residual-stress analysis. In amorphous systems, residual stress is governed primarily by constrained thermal contraction below the glass-transition or stress-freezing range. In semi-crystalline systems, thermal contraction and crystallization shrinkage act together, while their relative contributions are modified by reinforcement, cooling rate, local reheating, nucleation behavior, and post-process thermal history. Consequently, thermal-management strategies cannot be transferred directly between matrix classes without accounting for their different relaxation and crystallization behavior.

### 2.5. Printed Mesostructure, Porosity, and Anisotropy

FFF parts are not homogeneous solids. They contain inter-road and interlayer voids whose size, shape, and distribution depend on road overlap, layer height, extrusion multiplier, material viscosity, and processing conditions [94]. Their stiffness and strength are therefore strongly direction dependent [95,96,97,98,99,100]. Raster angle, air gap, and build orientation can change tensile strength by tens of percent and shift the dominant failure mode from in-road fracture to interfacial failure [43,95,101].

For reinforced feedstocks, flow-induced fiber alignment adds an additional level of anisotropy. Stiffness and the coefficient of thermal expansion become direction dependent, with longitudinal thermal expansion strongly suppressed by aligned fibers and transverse expansion remaining more matrix-dominated [9,15,102,103]. This anisotropic mesostructure is simultaneously a product of the deposition process and a governing input for residual-stress formation. It determines how shrinkage is constrained within individual roads, between adjacent roads, between layers, and at the part scale. Consequently, residual stress in printed reinforced thermoplastic composites must be interpreted as a multiscale phenomenon rather than as a homogeneous bulk stress state.

Several representative cases illustrate how mesostructure, porosity, and anisotropy affect residual-stress interpretation in material-extruded polymers and composites. In neat ABS FFF parts, the deposited roads form an anisotropic mesostructure with inter-road and interlayer voids; the resulting mechanical response depends strongly on raster angle, build orientation, air gap, and the continuity of bonding between adjacent roads [94,95,96,97,98,99,100,101]. This case represents the baseline condition in which residual stress is governed mainly by constrained thermal contraction and by the heterogeneous stiffness of the printed road network.

In short-fiber-reinforced systems, the mesostructure becomes more complex because fibers align preferentially with melt flow and the deposition direction. Carbon-fiber-reinforced ABS and PLA systems show that fiber alignment increases longitudinal stiffness and reduces shrinkage along the print path, but transverse and through-thickness regions remain more matrix-dominated and void-sensitive [9,10,15,31,104]. For SCF/PLA structural sections, geometry and fiber-induced anisotropy were shown to generate non-uniform stress distributions; square sections developed edge-concentrated residual stresses, whereas circular sections exhibited more uniform stress fields and lower warpage [34]. This example demonstrates that mesostructure and macroscopic geometry cannot be separated in residual-stress assessment.

Large-format carbon-fiber-reinforced extrusion provides a further typical case. In BAAM-scale CF/ABS systems, large bead dimensions, high short-fiber content, slow cooling, and anisotropic thermal expansion make the residual-stress state strongly dependent on bead-scale temperature history and fiber-orientation-dependent properties [15,16,17,66,67]. Although carbon fibers reduce visible distortion by lowering effective shrinkage and increasing stiffness, the retained internal stress may remain significant. Therefore, large-format printed composites require simultaneous interpretation of thermal history, bead geometry, porosity, fiber orientation, and measured distortion.

## 3. Formation Mechanisms of Residual Stresses in FFF

### 3.1. Basic Assessment Framework for Residual Stress and Distortion

In material extrusion additive manufacturing, residual stress and distortion should be assessed as related but not equivalent quantities. Residual stress is an internal self-equilibrated stress state generated by constrained thermal contraction, crystallization shrinkage, reinforcement-induced anisotropy, and local mechanical constraint during deposition and cooling. Distortion, including warpage, corner lift, curvature, and dimensional deviation, is the externally visible deformation that appears when part stiffness, geometry, build-platform adhesion, and stress release allow the stored stress to generate macroscopic displacement. Therefore, low distortion does not necessarily indicate low residual stress, especially in reinforced thermoplastic composites, where fibers and fillers may reduce visible warpage by increasing stiffness while retaining higher internal stress [29,30,31,83].

A basic assessment of residual stress and distortion in material extrusion should therefore combine three levels of evidence. The first level is direct or semi-direct residual-stress or residual-strain measurement, including hole drilling, layer removal, curvature reconstruction, and embedded fiber Bragg grating sensors [30,42,84,105,106]. The second level is deformation-based assessment, including warpage, corner lift, flatness deviation, deflection monitoring, and digital image correlation, which captures the structural response after constrained cooling and release [35,36,48,67,107]. The third level is supporting process and material characterization, including thermal history, crystallinity, porosity, fiber orientation, interlayer bonding, and build-platform constraint, because these variables define the stress-generating eigenstrains and the mechanical boundary conditions [26,27,28,34,90,94,104]. For reinforced thermoplastic composites, the most reliable assessment is therefore not a single warpage value, but a combined interpretation of residual-stress measurement, distortion metrology, thermal history, and mesostructural characterization.

### 3.2. Process Principle and Thermal Character

The origin of residual-stress in FFF is the constrained, non-uniform contraction of material that solidifies at various times and positions. A road leaves the nozzle at the melt temperature (e.g., 230–260 °C for ABS and up to 470 °C for PEEK) and cools toward the ambient temperature with characteristic times of the order of seconds [26,27]. While the polymer is above its glass transition (or, for semi-crystalline polymers, above the crystallization range), it can flow and relax. Once it passes below this “stress-freezing” threshold, further thermal contraction is elastic and constrained by the substrate, generating stress [29,30]. Deposition of the next road or layer reheats the interface—often above Tg in the immediately adjacent material [62,69] which partially anneals existing stress locally but also re-establishes the cycle, so that each material point experiences several freeze-reheat-freeze events of decaying amplitude [65,105]. The cumulative result is a layerwise stress buildup with pronounced gradients through the part height and concentrations near free edges and the first layer [47,48]. As illustrated schematically in Figure 1, residual stress development in FFF should be interpreted as a repeated freeze–reheat–freeze sequence rather than as a single cooling event. A newly deposited road first contracts under constraint, subsequent deposition partially reheats and relaxes the previously solidified material, and final cooling locks the remaining strain into the part. The resulting stress therefore depends not only on the initial temperature drop but also on the full thermal history, layer timing, local reheating intensity, and constraint imposed by the substrate and surrounding roads.

### 3.3. Constrained Shrinkage: Thermal and Crystallization Contributions

Two volumetric mechanisms contribute to stress buildup. First, thermal contraction: The volumetric shrinkage between the stress-freezing temperature and room temperature, proportional to the CTE and the temperature interval. Material property sensitivity studies identify CTE as the dominant material parameter governing FDM warpage [108]. Second, crystallization shrinkage: Semi-crystalline polymers contract additionally upon crystallization, with the developed crystallinity itself depending on the local cooling history [32,89,90]. Process simulations that neglect crystallization systematically underpredict distortion in semi-crystalline systems, which models coupled with crystallization kinetics [33,93,109] (Section 5.3). The classic analogy is the bimetallic strip: a newly deposited hot layer bonded to a colder substrate develops a moment upon cooling. Analytical warpage models for FDM are built directly on this principle [29,35].

### 3.4. Typical Stress Distributions and Geometry Effects

Measured and simulated stress fields in printed parts share recurring features. Hole-drilling measurements on ABS parts show tensile residual stresses at the top surface and lower, partly compressive stresses near the bottom, with magnitudes depending on the deposition path [30,110]. Process simulations of simple prisms locate stress concentrations at the corners of the first layer (bed constraint) and at free edges [48,49,111]. Geometry matters: in short carbon-fiber PLA tubes, square cross-sections develop edge-concentrated stresses with maxima around 108 MPa, whereas circular cross-sections exhibit nearly uniform distributions and smaller warpage [34]. Within a layer, the raster pattern redistributes stress: aligned (0°) rasters concentrate longitudinal stress, 90° rasters shift it transversely, and alternating patterns produce more balanced fields. This dependence has been systematically mapped by layer-resolved FEM studies [47,112]. Because the platform constrains the part during printing, a substantial fraction of the stress manifests as distortion only upon removal. Simulations that include the detachment stage reproduce this stress release and the associated springback [49].

**Figure 2:**
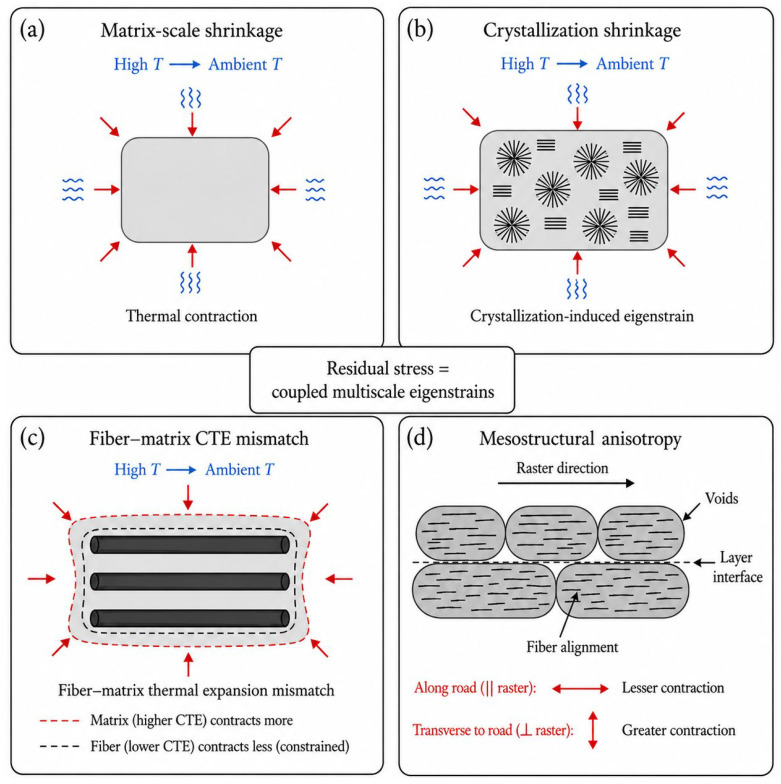
Schematic illustration of the coupled multiscale eigenstrain sources governing residual-stress development in material-extruded reinforced thermoplastic composites: (**a**) matrix-scale thermal contraction, (**b**) crystallization-induced shrinkage in semi-crystalline matrices, (**c**) fiber–matrix coefficient-of-thermal-expansion mismatch, and (**d**) mesostructural anisotropy associated with raster orientation, fiber alignment, voids, and interlayer interfaces.

The mechanisms summarized in Figure 2 can be linked to several typical material and process cases. In neat amorphous polymers such as ABS, residual stress is governed primarily by constrained thermal contraction below the glass transition temperature; hole-drilling and deformation studies show that this produces layer-dependent tensile and compressive stress gradients that vary with deposition path and platform constraint [30,31,110]. In semi-crystalline high-temperature polymers such as PEEK and PAEK, the same thermal-contraction mechanism is coupled with crystallization shrinkage, so chamber temperature, cooling rate, and post-deposition crystallization strongly affect warpage, delamination, and final residual stress [32,38,39,68]. In short-fiber-reinforced thermoplastics, such as SCF/PLA or CF/ABS, the aligned fibers reduce shrinkage along the print direction but increase orthotropic stiffness and constraint; therefore, stress concentrations depend on fiber orientation, raster architecture, and part geometry [9,31,34,104]. In continuous-fiber material extrusion, the deposited fiber path acts as a local mechanical constraint, making residual strain strongly dependent on fiber trajectory, lay-up, curvature, and interfacial bonding [12,13,14,84]. These representative cases show that residual-stress formation in FFF cannot be assigned to a single mechanism; it is instead governed by the superposition of thermal contraction, crystallization shrinkage, fiber–matrix mismatch, mesostructural anisotropy, and process-imposed constraint.

### 3.5. Composite-Specific Mechanisms

Reinforcement changes every term of the stress balance. Fibers and rigid fillers reduce the effective CTE along their orientation, directly reducing the driving force for stress and warpage. This mechanism enables low-distortion large-format printing with carbon-fiber contents of 13–20 wt.% [9,15,66]. Experimentally, CNT and short-carbon-fiber-filled ABS show markedly lower shrinkage and residual deformation than neat ABS [31], and CNT networks improve the thermal-dimensional stability of printed ABS [7,82]. Anisotropy: because fibers align with the deposition direction [9,104], both stiffness and CTE become strongly orthotropic. The mismatch between road longitudinal and road transverse contraction generates inter-road and interlaminar stresses analogous to ply-level stresses in laminates [44,67]. Embedded FBG measurements in continuous-fiber parts confirm orientation-dependent residual strain magnitudes [84]. Microscale stresses: As in conventional composites, the CTE mismatch between fiber and matrix produces fiber–matrix (micro) residual stresses superimposed on the road and part-level (meso/macro) stresses [44]. Porosity: Fiber loading typically increases void content [9,31], which relaxes some constraint but degrades strength and complicates measurement and modeling (Section 4.4 and Section 5.7). Crystallinity interactions: Fillers and fibers act as nucleating agents and alter crystallization kinetics, coupling reinforcement content to the crystallization shrinkage term [34,90]. The residual stress state of a printed reinforced thermoplastic composite can therefore be interpreted as the superposition of four coupled eigenstrain sources: thermal contraction, crystallization shrinkage, fiber–matrix thermal expansion mismatch, and mesostructural anisotropy. Their relative importance depends on matrix type, reinforcement architecture, raster strategy, cooling rate, chamber temperature, porosity, and post-process thermal history. This multiscale coupling explains why reduced macroscopic distortion does not necessarily imply reduced internal stress in reinforced feedstocks. The coupled eigenstrain sources are summarized in Figure 2.

**Figure 3:**
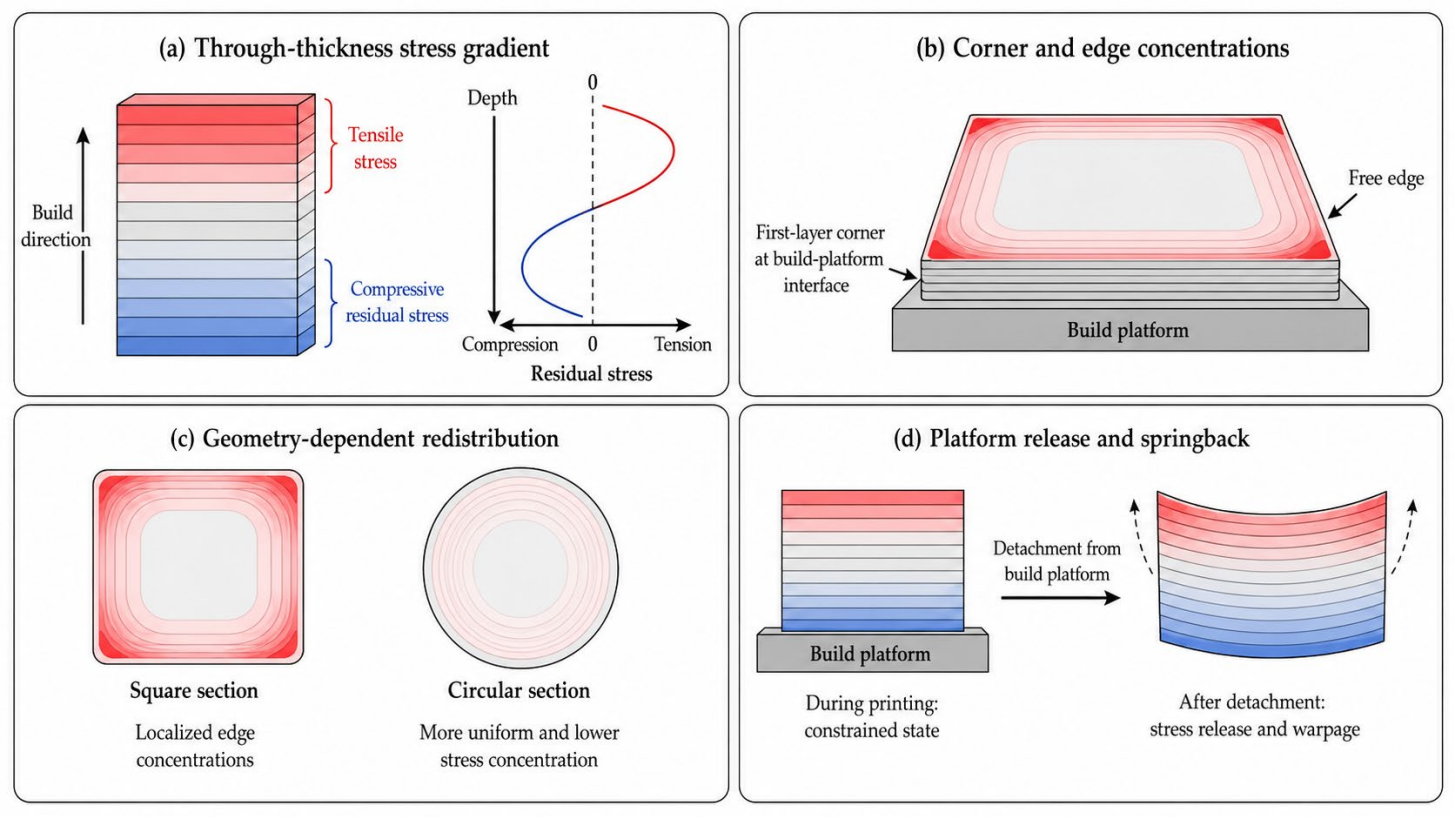
Schematic representation of characteristic residual-stress distributions and deformation modes in material extrusion additive manufacturing: (**a**) a representative through-thickness tensile–compressive residual-stress gradient, (**b**) stress concentrations at the first-layer corners adjacent to the build platform and at free edges, (**c**) representative geometry-dependent stress redistribution in square and circular sections, and (**d**) residual-stress release, springback, and warpage following detachment from the build platform.

The coupled eigenstrain sources summarized in Figure 2 manifest at the part scale as characteristic through-thickness residual-stress gradients, first-layer corner and free-edge stress concentrations, geometry-dependent stress redistribution, and deformation following detachment from the build platform, as schematically illustrated in Figure 3 [30,34,47,48,49,110,111,112]. These distributions should be interpreted as representative rather than universal, because their magnitude, sign, and spatial extent depend on the matrix and reinforcement system, raster architecture, part geometry, thermal history, build–platform constraint, and measurement or simulation conditions.

These characteristic distributions provide the structural link between the stress-formation mechanisms discussed above and the dimensional, interfacial, and mechanical consequences considered in the following section.

### 3.6. Consequences—And Deliberate Exploitation

The engineering consequences of residual stress in printed composites span several scales. At the part scale, warpage and corner lift compromise dimensional tolerance and can abort builds through bed detachment [35,36,37,107]. At the interface scale, residual tension superimposed on weak interlayer bonds promotes delamination and cracking, notoriously in PEEK printed in cold environments [32,38,39]. At the material scale, tensile residual stresses reduce effective strength while compressive stresses can increase it, as shown by combined bridge curvature/FEM studies on printed PLA [42], and stress relaxation over time contributes to dimensional instability. Conversely, the predictability of process-induced stress enables functional use: flat composite sheets printed with tailored raster patterns morph into prescribed 3D shapes upon heating, the basis of FFF-based 4D printing demonstrated for PLA, ABS, and wood–plastic composites [45,46,47]. Quantitative understanding of formation mechanisms is thus equally relevant for suppressing and for programming residual stresses.

## 4. Measurement and Characterization Methods

**Figure 4:**
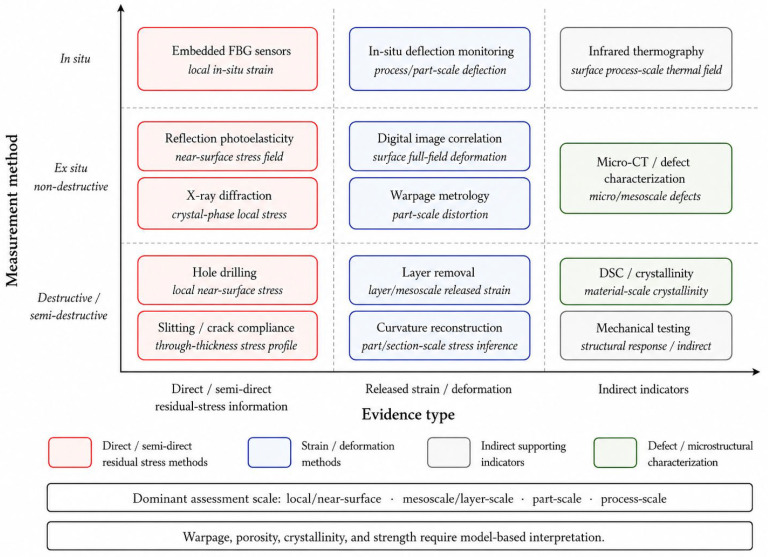
Classification of residual-stress measurement and characterization methods.

Residual-stress measurement techniques developed for metals and laminated composites must be adapted to printed polymers, which are compliant, viscoelastic, porous, orthotropic, and often amorphous. Table 4 compares the principal methods; the following subsections review them in detail. The classification of measurement approaches is shown in Figure 4.

### 4.1. Relaxation (Destructive and Semi-Destructive) Methods

From a comparative perspective, the methods in Table 4 should not be interpreted as equivalent alternatives. Relaxation-based methods, including hole drilling, layer removal, sectioning, slitting, and curvature reconstruction, provide the most direct access to residual stress or released strain, but they are destructive or semi-destructive and require elastic-property assumptions, calibration functions, and, for reinforced materials, orthotropic mechanical models. Embedded FBG sensors are the most suitable for in situ monitoring because they capture the evolution of strain during deposition and cooling; however, they provide local rather than full-field information and require temperature compensation. DIC and in situ deflection monitoring provide full-field or global deformation data and are valuable for model validation, but they generally require inverse modeling to obtain stress values. Diffraction and photoelastic methods are material-specific: X-ray diffraction is limited to crystalline phases, whereas transmission or reflection photoelasticity is mainly suitable for transparent polymers or near-surface comparative assessment. Warpage metrology is the most practical industrial indicator, but it is indirect and can be misleading in reinforced composites because increased stiffness may reduce visible distortion while retaining higher internal stress.

The technical development of relaxation-based residual-stress methods in printed polymers and composites can be interpreted as a transition from simple strain-release measurements toward hybrid, full-field, and material-specific inverse approaches. In conventional hole drilling, the main innovation has been the replacement or supplementation of bonded strain gauges by optical displacement measurements such as ESPI and DIC, which are better suited to compliant, rough, and heterogeneous FFF surfaces [30,110,113,116]. For reinforced composites, a second important development is the use of orthotropic finite-element calibration rather than isotropic calibration constants, because fiber orientation, porosity, and layer architecture alter the released-strain field [114,115,116,117,128]. Layer-removal techniques developed from classical one-dimensional stress-relief approaches for polymers and orthotropic laminates [118,119] toward FFF-specific layer-resolved deformation mapping combined with DIC and microstructural characterization [31]. Curvature and bridge-type methods link optically measured released distortion to inverse FEM reconstruction [42], whereas crack-compliance and slitting methods remain promising for through-thickness stress profiling in layered composite structures [120,121,122]. The principal innovation is therefore not a single new destructive test, but the integration of controlled stress release with full-field optical measurement, orthotropic calibration, and model-based reconstruction.

#### 4.1.1. Hole Drilling

The incremental hole-drilling method (HDM), standardized for isotropic metals, infers residual stress from strains released by drilling a small hole. Casavola et al. pioneered its application to FDM parts, combining hole drilling with electronic speckle pattern interferometry (ESPI) to avoid gauge installation on compliant ABS surfaces, and demonstrated stacking-sequence-dependent residual stresses [30,110,117]. For fiber composites the isotropic formalism is invalid: orthotropic adaptations compute calibration coefficients by finite element analysis of the actual lay-up [114,115,128]. Studies on CFRP laminates further show that porosity alters the calibration and hence the inferred stresses—a caveat that transfers directly to void-rich printed composites [117]. Recent work replaces strain gauges with digital image correlation around the hole, easing application to printed surfaces [116], and combines photoelastic localization with hole-drilling quantification on transparent PLA [50].

#### 4.1.2. Layer Removal, Sectioning, and Curvature

Layer removal techniques, long used for bulk polymers [118] and adapted to orthotropic laminates [119], translate naturally to layered FFF parts. Zhang et al. combined sectioning with digital image correlation and thermal shrinkage recovery tests to map residual deformation in ABS, CNT/ABS, and short CF/ABS specimens, complemented by micro-CT porosity analysis [31]. Curvature-based approaches measure the deflection of asymmetric or released specimens: the bridge curvature method combined with optical scanning and FEM reconstruction recovers the stress state of printed PLA parts and correlates it with strength [42]. Crack compliance/slitting methods, established for layered composites [120,122], have seen little FFF-specific application and represent an opportunity, particularly for continuous-fiber parts where through-thickness profiles are of interest.

### 4.2. Non-Destructive and In Situ Methods

#### 4.2.1. Embedded Fiber Bragg Gratings (FBGs)

Optical fibers with Bragg gratings embedded between layers during printing record strain (and, with compensation, temperature) throughout the build and afterwards. Kantaros and Karalekas first quantified process-induced residual strains in FDM ABS this way [106]; subsequent work mapped in situ strain and temperature distributions [105], extended the methodology to continuous-fiber composites [84], and used FBGs to measure orientation-dependent CTE of printed materials—a key model input [102,103]. Embedding the fiber does not significantly degrade part durability, supporting FBGs as in-service monitors as well [123].

#### 4.2.2. Full-Field Optical Methods

Digital image correlation provides non-contact, full-field displacement data during and after printing. Reviews document its growing use for distortion and residual-stress assessment in AM [124], and large-scale composite AM has been monitored in situ with DIC exploiting the natural surface texture of deposited beads [125]. In situ deflection monitoring of instrumented substrates offers a simple scalar proxy for stress buildup and has been used to validate process FEA [48]. Infrared thermography, while not a stress measurement, supplies the thermal history needed to interpret and validate all stress models [26,66,87].

#### 4.2.3. Diffraction, Photoelasticity, and Related Techniques

X-ray diffraction, the workhorse for metals, applies only to crystalline phases. In polymers it is limited to semi-crystalline fractions and embedded crystalline fillers and is rarely quantitative for printed parts [42]. Photoelasticity is applicable to transparent amorphous polymers and has been used for injection-molded parts and, recently, for rapid qualitative mapping of stress concentrations in printed PLA, combined with hole drilling for quantification [50,127]. Reflection photoelasticity extends this principle to opaque printed composites by using a reflective photoelastic coating bonded to the specimen surface. Recent work on aramid fiber-reinforced ASA specimens produced by material extrusion showed that PhotoStress can visualize and compare near-surface residual-stress relaxation under controlled measurement conditions, particularly when combined with an abrasive water-jet relief cut as a repeatable stress release feature [129]. The method is best interpreted as comparative and semi-quantitative unless coating calibration, fringe order evaluation, surface preparation, and stress-release geometry are rigorously controlled. Raman spectroscopy offers local stress sensitivity in principle but lacks validated FFF applications.

### 4.3. Indirect Indicators: Warpage Metrology

Because warpage is the most visible manifestation of residual stress, standardized distortion metrics serve as practical comparators. Corner lift measurements on rectangular plates [36,70], image-based corner height indices for PEEK [107], flatness deviation from 3D scanning [42], and LVDT bottom surface profiles on BAAM walls [67] all quantify the integrated effect of the stress field. Analytical relations between part dimensions and warpage allow such data to be reduced to comparable parameters [29,35]. Caution is needed when comparing studies: warpage reflects stress and stiffness and constraint, so identical distortions can correspond to substantially different stress magnitudes, notably in fiber-filled materials, where stiffening can mask stress increases [83].

### 4.4. Critical Comparison and Sources of Uncertainty

For printed composites, no single method is sufficient. Relaxation methods provide quantitative stress values but rely on elastic-constant and calibration assumptions that are challenged by orthotropy, inter-road voids, and surface roughness [114,117]. Polymer viscoelasticity allows stresses to relax between manufacturing and measurement, so the measured state depends on specimen age and storage temperature—an effect documented for thermoplastic composites in general [130] and rarely controlled in FFF studies. FBGs offer unmatched in situ insight but measure strain at discrete embedded points, perturb the local mesostructure, and require careful temperature–strain decoupling [105,123]. Full-field optical methods capture distortion, not stress, and require model-based inversion. Related standards assume isotropic, homogeneous materials; no dedicated standard exists for residual-stress measurement in printed polymers or printed composites, impeding interlaboratory comparability [30,42]. Best practice therefore combines at least one relaxation method, one in situ sensor, and distortion metrology, together with full reporting of material anisotropy, porosity, and elapsed time after printing. Defect characterization should be treated as a supporting requirement rather than as an optional supplement. Voids, incomplete inter-road fusion, interlayer delamination, local fiber misalignment, and void clustering modify the local stiffness and constraint conditions and may therefore change both the actual residual-stress field and the apparent stress obtained from relaxation measurements. Micro-CT, thermography, ultrasonic inspection, optical methods, and self-sensing fiber systems are therefore valuable companions to residual-stress measurement in reinforced material extrusion composites [131]. Without such information, differences attributed to material formulation or process parameters may partly reflect changes in defect population.

Therefore, method selection should be based on the intended level of evidence. Direct stress quantification requires relaxation-based or sensor-based methods with material-specific calibration, whereas process comparison and industrial monitoring may rely on distortion, thermal, or optical indicators only when their indirect character is explicitly acknowledged. For printed reinforced thermoplastics, the strongest experimental strategy is a combined workflow linking residual-stress measurement, full-field deformation, thermal history, and defect characterization.

**Figure 5:**
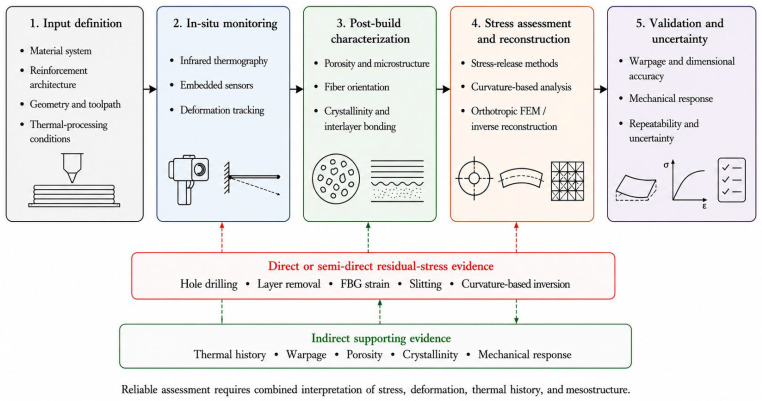
Integrated workflow for residual-stress assessment in material-extruded reinforced thermoplastic composites. The workflow links input definition, in situ monitoring, post-build characterization, stress assessment and reconstruction, and validation with uncertainty evaluation. Direct or semi-direct residual-stress evidence is distinguished from indirect supporting evidence based on thermal history, distortion, porosity, crystallinity, and mechanical response.

Based on the complementary capabilities and limitations of the reviewed methods, Figure 5 presents an integrated workflow for residual-stress assessment in material-extruded reinforced thermoplastic composites. The workflow combines material and process definition, in situ monitoring, post-build characterization, stress-release measurements, orthotropic model-based reconstruction, and independent validation [30,42,84,131]. It distinguishes direct or semi-direct residual-stress evidence from indirect supporting indicators because reduced warpage or improved process-quality metrics do not uniquely demonstrate a lower retained residual-stress state [31,83].

The proposed workflow emphasizes that reliable residual-stress assessment requires convergence between stress-release or embedded-strain evidence, process monitoring, mesostructural characterization, and model-based interpretation rather than reliance on a single distortion or mechanical-performance metric.

## 5. Modeling and Simulation

Modeling residual stress in FFF requires resolving (or judiciously homogenizing) a moving heat source, sequential material addition, temperature-dependent and possibly crystallinity-dependent constitutive behavior, and the mechanical constraint of the build platform. Table 5 summarizes representative studies, with particular attention to the type and level of experimental validation. This section reviews the main model families.

A critical distinction in Table 5 is the level of experimental validation. Temperature-field validation confirms the heat-transfer part of a process model, whereas warpage or distortion validation confirms only the integrated structural response after constrained cooling and release. Direct residual-strain or residual-stress validation is more demanding and remains less common, particularly for reinforced and continuous-fiber systems. Experimentally validated models are therefore most reliable when they combine thermal measurements, post-print distortion or deflection data, and, where possible, residual-strain or residual-stress measurements. This distinction is important because reinforced composites may show reduced visible distortion while retaining higher internal stress in a stiffer structure.

### 5.1. Analytical Models

**Figure 6:**
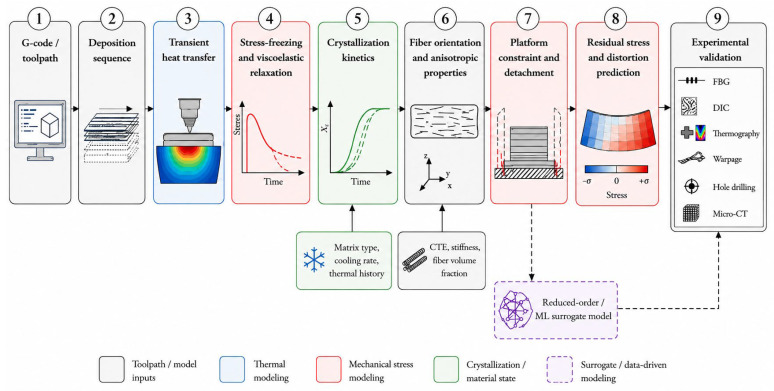
Modeling workflow for residual-stress prediction.

The earliest quantitative treatments idealize the part as a stack of prestressed layers. Wang et al. derived a closed-form warp deformation model from layerwise thermal contraction, relating distortion to the number of layers, section geometry, and the temperature interval below the stress-freezing point [29]. Armillotta et al. combined designed experiments with a laminate-type analytic model that captures the dependence of warpage on part length, width, and thickness for ABS prisms, providing practical design rules [35]. Analytical models remain valuable as sanity checks and for slicing-level heuristics, and modern FEM studies still benchmark against them [138], but they cannot represent raster patterns, geometry effects, or crystallization. A general modeling workflow is illustrated in Figure 6.

### 5.2. Sequential Thermo-Mechanical Finite Element Simulation

The dominant numerical approach reproduces the deposition sequence by element activation (“birth and death” or quiet elements) in a transient thermal analysis whose temperature fields drive a subsequent (or staggered) mechanical analysis. Zhang and Chou established this framework for FDM, simulating road-by-road deposition and conducting parametric distortion studies of speed, layer thickness, and road width [132,133]. Cattenone et al. systematically analyzed the impact of meshing strategy, time step, and material model on distortion prediction, validating against printed ABS parts [111]. Subsequent contributions improved fidelity and efficiency: G-code-driven event series automate activation from the actual toolpath [49,139], voxel-based local refinement and adaptive schemes reduce cost [137,140], quiet element formulations enable in situ validated deflection prediction [48], and fully resolved front-tracking simulations capture bead-scale solidification stresses [141]. Validation studies report distortion prediction deviations of roughly 10% when temperature-dependent properties and the platform detachment stage are included [49,137]. Layer-resolved FEM has also been used to map raster patterns dependent on stress distributions, including wood–plastic composites and 4D-printing design [47,112]. El Moumen et al. extended the framework explicitly to polymer composites, simulating temperature and residual-stress fields during printing of reinforced thermoplastics [134].

### 5.3. Crystallization-Coupled and Viscoelastic Models

For semi-crystalline matrices, accurate residual-stress prediction requires coupling heat transfer to crystallization kinetics and to a constitutive law whose stiffness and specific volume evolve with crystallinity. The Purdue/ORNL school developed this methodology for fiber-reinforced high-temperature polymers in extrusion deposition AM: thermoviscoelastic constitutive modeling [85], fusion-bonding/crystallization submodels [86,109], and validated process simulations of 50 wt.% CF/PPS [33]. Samy et al. implemented crystallization kinetics with a generalized Maxwell viscoelastic model in COMSOL for polypropylene, quantifying the influence of printing conditions, ambient temperature, and raster pattern on distortion and stress, and contrasting amorphous and semi-crystalline behavior [93,112,135,136]. Jiang et al. coupled crystallinity evolution to a multi-physics model of short CF/PLA structural sections and validated stress and warpage trends experimentally [34]. Neglecting viscoelastic relaxation overestimates locked-in stress, particularly for slow-cooling large parts. Thermoviscoelastic formulations therefore matter most for BAAM-scale components [16,85].

### 5.4. Composite-Specific Inputs: Fiber Orientation and Anisotropic Properties

Fiber-reinforced simulations require local anisotropic thermo-elastic properties, which depend on flow-induced fiber orientation. Nozzle and deposition flow simulations using orientation tensor methods predict the orientation state as a function of nozzle geometry, swell, and printing conditions [104,142,143,144], and orientation fields measurably change predicted deformation [145]. At the industrial scale, ORNL’s de-homogenization approach maps fiber orientation-dependent properties (with TMA measured anisotropic CTE) onto BAAM walls, with IR and LVDT validation of temperature and distortion [67]. The same philosophy underpinned the simulation of the first full-size BAAM printed car [16]. Thermal submodels for large-format depositions, such as the 1D transient analysis of BAAM walls, set the layer time limits within which distortion remains acceptable [66,87].

### 5.5. Commercial Tools and Accessibility

Commercial chains have made process simulation accessible to non-specialists. Digimat-AM has been used to predict residual stress and warpage of ABS, PEI, PA6, glass-fiber ABS, and chopped CF PA6 parts as functions of printing parameters [83,88,146,147,148,149]. ANSYS-based thermomechanical models served Taguchi-style warpage optimization [138], Abaqus underlies both academic frameworks [49,111] and the Purdue Additive3D toolchain [33], and COMSOL hosts the crystallization-coupled PP models [93]. Reviews of FFF numerical simulation catalog these approaches and their maturity [25,51,150,151,152].

### 5.6. Reduced-Order, Data-Driven, and Hybrid Approaches

Reduced-order models are increasingly relevant for large-format polymer and composite extrusion, where fully resolved road-by-road continuum simulations are computationally expensive. Beam-based finite-element formulations represent deposited beads using structural elements while retaining sufficient kinematic information to predict global residual stresses and displacements [153]. Such approaches cannot replace high-fidelity thermo-viscoelastic process simulations, but they provide a practical intermediate level between detailed physics-based modeling and process planning tools. For reinforced systems, reduced-order models should still retain the essential anisotropic inputs: deposition direction, temperature-dependent stiffness, anisotropic CTE, platform constraint, and the release step after detachment.

Machine learning enters residual-stress management chiefly through distortion prediction and in-process detection. Multivariate thermal time series classified by k-nearest neighbors forecast warping during printing [154]. Convolutional neural networks detect warp onsets from camera images with >99% reported accuracy and enable closed-loop response [155]. Multi-head networks generalize error detection and correction across printers and materials, including warp recognition and parameter correction [156,157]. Surrogate models trained on FEM campaigns to predict quantitative stress fields for composite FFF remain scarce—an identified research opportunity (Section 8).

### 5.7. Limitations of Current Predictive Models

Despite substantial progress, current predictive models of residual stress in material extrusion remain limited by several factors. First, many simulations are validated against temperature fields, warpage, or global distortion rather than against direct residual-stress or residual-strain measurements [49,111,137,148]. Such validation is useful but incomplete, particularly for reinforced composites, where increased stiffness may suppress visible deformation while retaining higher internal stress [83]. Second, reliable prediction requires temperature-dependent and, for semi-crystalline polymers, crystallinity-dependent material data, including modulus, coefficient of thermal expansion, relaxation behavior, crystallization kinetics, shrinkage, and specific-volume evolution [33,85,86,93,135,136]. These data are often unavailable for commercial reinforced filaments and are commonly approximated from neat-polymer or bulk-composite values.

Third, the mesostructure of printed composites is difficult to represent with sufficient fidelity. Fiber orientation, fiber length degradation, inter-road porosity, incomplete fusion, local void clustering, and fiber-matrix interfacial quality all affect local stiffness and thermal contraction, but they are frequently homogenized or omitted [31,67,104,131,142,143,144,145]. Fourth, the mechanical boundary conditions during printing are difficult to model accurately. Build-platform adhesion, partial detachment, brims, rafts, local contact loss, and the release step after part removal can strongly influence the final stress and distortion state [37,49]. Finally, fully resolved road-by-road thermo-viscoelastic simulations remain computationally expensive, particularly for large-format and continuous-fiber structures [66,87,153]. Therefore, predictive models should be interpreted according to their validation level: thermal validation supports the heat-transfer model, distortion validation supports the integrated deformation response, but direct residual-strain or residual-stress validation is required before quantitative stress fields can be considered reliable.

## 6. Effects of Process Parameters and Material Composition

The parameter dependence of residual stress and warpage has been probed experimentally and numerically across materials; Table 6. condenses the evidence. Headline trends are summarized below with their mechanistic interpretation.

### 6.1. Thermal Parameters

Every measure that reduces the temperature gradient between deposited material and its surroundings reduces locked-in stress. Raising ambient/chamber temperature flattens the cooling curve and lowers distortion, with simulations quantifying the effect for both amorphous and semi-crystalline polymers [135,136] and PEEK practice demanding heated chambers as a precondition for crack-free parts [32,38,39]. Auxiliary local heating of the deposition zone in CF/ABS reduced corner warpage from 12.3 mm to about 1 mm while simultaneously improving interlayer strength—among the largest mitigation effects reported [70]. Infrared preheating achieves analogous interface benefits at BAAM scale [69]. Bed temperature controls first-layer adhesion and the constraint under which the part cools. Optimized platform adhesion suppresses warpage-induced detachment for high shrinkage PP [37]. Nozzle temperature acts more weakly and non-monotonically: hotter deposition improves bonding and local annealing but enlarges the contracting temperature interval [88,101]. Vacuum-assisted material extrusion is an emerging thermal-management route for high-temperature matrices such as PEEK and PAEK composites. By suppressing convective heat transfer, vacuum printing slows heat dissipation and can extend the time available for interlayer bonding, crystallization, and stress relaxation. In PEEK, printing under low pressure was shown to increase crystallinity, improve neck growth and molecular diffusion between adjacent beads and layers, reduce voids, and improve warping deformation compared with atmospheric printing [72]. Similar benefits have been reported for 30 wt.% short carbon-fiber-reinforced PEEK, where printing at 0.5 mbar increased flexural stress and elastic modulus while reducing porosity compared with atmospheric conditions [73]. These studies provide strong indirect evidence that reduced convective cooling can mitigate stress-driving thermal gradients, although direct residual-stress measurements under vacuum remain scarce.

### 6.2. Kinematic and Geometric Parameters

Printing speed raises stress and porosity: faster deposition shortens reheating windows and increases cooling rates, with measurably larger shrinkage and residual deformation in ABS and its composites [31], and simulations confirm speed-dependent stress [135]. Layer thickness affects both thermal mass and the number of thermal cycles. Numerical studies find thicker layers reduce residual stress (fewer cycles, slower cooling) at a surface quality cost [133,146]. Raster angle and infill pattern redistribute the stress field. A 0°/90° alternation and concentric patterns repeatedly emerge as low-stress options, with concentric infill minimizing residual stress in Digimat studies [47,112,147]. Infill density increases both stiffness and locked-in stress while reducing distortion [83]. Part geometry remains decisive: larger in-plane dimensions warp more [29,35], corners and edges concentrate stress relative to rounded sections [34,48], and first-layer corners are the canonical hot spots [48,111].

### 6.3. Reinforcement Type and Content

Particulate and short-fiber reinforcements trade lower distortion for higher locked-in stress. CNT and short carbon fibers cut shrinkage and residual deformation of ABS markedly, at the price of higher porosity [7,31]. Expanded perlite reduced PP warpage by roughly a quarter [81]. Small glass spheres plus chamber temperature control improved PP dimensional accuracy [75] and 13–20 wt.% CF underlies warp-free large-format printing [9,15,17]. Simulation of glass-fiber ABS shows the complementary side: increasing fiber ratio and infill decreases distortion but increases the thermal residual stress retained in the stiffer part [83]; distortion-based assessments alone therefore mislead (Section 4.3). For continuous-fibers systems, quantitative residual-stress data are scarce. FBG-instrumented studies demonstrate orientation- and lay-up-dependent residual strains [84], and modeling work confirms strong sensitivity to fiber path [145,152], but systematic stress characterization of continuous-fiber FFF parts is a conspicuous gap. Recent work on continuous carbon-fiber-reinforced PEEK further illustrates this gap. Vacuum printing has been reported to improve the flexural performance and microstructural quality of continuous-fiber PEEK composites by reducing convective heat transfer, improving interfacial bonding, and reducing void formation [158]. Infrared preheating combined with a high fiber volume fraction has also been shown to improve interlaminar bonding and mechanical performance in continuous CF/PEEK material extrusion [159]. These results are highly relevant to residual-stress control because vacuum and local reheating directly alter the thermal boundary conditions under which stresses are generated and frozen in. Nevertheless, they should be interpreted primarily as indirect evidence unless combined with direct residual strain or residual stress measurements. Natural fiber and wood fillers reduce density and CTE but introduce hygroscopic swelling as an additional eigenstrain source, exploited deliberately in hygromorphic actuators [76] and relevant to the stress state of WPC prints [47]. Recycled-carbon-fiber-filled systems also show improved dimensional and mechanical stability. For rCF/ABS, improved dimensional stability during post-process annealing indicates filler-stabilized deformation during stress relief [160]. Studies of rCF/PLA filaments additionally demonstrate effective mechanical reinforcement and altered physical properties, although direct residual-stress measurements remain unavailable [161,162].

### 6.4. Matrix Selection and Crystallinity

Across studies, semi-crystalline matrices carry the additional crystallization shrinkage term (Section 3.3), making them more warp-prone for equal CTE. PP and PEEK are canonical difficult cases [38,81,92], whereas amorphous ABS, PETG, and PEI respond primarily to thermal-gradient management [88]. Within semi-crystalline systems, the printing temperature-dependent crystallinity couples mechanical properties and stress state [32,90,163], and slow-crystallizing PAEK chemistries demonstrate that matrix formulation itself is a stress-mitigation lever [68]. The available quantitative evidence is summarized in Table 7. The table distinguishes direct residual-stress evidence from indirect indicators such as warpage, shrinkage, porosity, crystallinity, and mechanical performance, because these quantities are not mechanically equivalent in reinforced composites.

Overall, the comparison confirms that reinforcement generally reduces visible distortion by lowering effective shrinkage and increasing stiffness. However, glass-fiber-reinforced ABS and related simulations show that lower warpage can coexist with higher retained thermal residual stress, confirming that distortion-based assessment alone is insufficient for reinforced composites.

In addition to reinforcement type and content, fiber orientation and raster architecture strongly influence the residual-stress field because they determine the local stiffness, coefficient of thermal expansion, shrinkage direction, and constraint between adjacent roads and layers. The main orientation-dependent effects are summarized in Table 8.

This comparison shows that fiber orientation affects residual stress not only through reinforcement stiffness but also through the directionality of thermal contraction. In short-fiber systems, flow-induced alignment reduces shrinkage along the road direction but leaves transverse and through-thickness contraction more matrix dominated. In continuous-fiber systems, the deposited fiber trajectory becomes a local mechanical constraint, so residual strain and distortion are governed by path direction, curvature, fiber tension, and interfacial quality. Therefore, orientation should be treated as a process-defined material variable rather than only as a geometric toolpath parameter.

### 6.5. Synthesis

Ranking the influences supported by multiple independent sources, 1 is the material system (CTE, crystallization behavior, reinforcement) [15,83,108], 2 is the thermal environment (chamber/bed/local heating) [70,136], 3 is part geometry and size [34,35], and 4 is the deposition strategy (raster, infill, speed, layer thickness) [31,47,88]. Apparent contradictions between studies usually dissolve once the response variable is identified as distortion versus stress and once constraint conditions (bed adhesion, brims, detachment before/after measurement) are accounted for [49,83].

## 7. Mitigation and Control Strategies

Mitigation measures act on the three ingredients of residual stress: the magnitude of constrained shrinkage, the temperature gradients during deposition, and the stress already locked into the finished part. Table 9 maps strategies to mechanisms and quantified outcomes.

### 7.1. In-Process Thermal Management

Heated build chambers and beds remain the baseline measure, mandatory for high-temperature polymers [32,38] and beneficial across materials [136]. Localized pre-deposition heating goes further by reheating only the bonding interface: laser pre-deposition heating raised ABS interlayer bond strength by ~50% [71], infrared lamp preheating improved BAAM CF/ABS interlayer strength [69], and an auxiliary heating plate reduced CF/ABS warpage by an order of magnitude [70]. Ultrasonic vibration during deposition offers an athermal alternative, improving interlayer adhesion of ABS by up to ~10% and tensile performance by ~11–17% [164,165]. Its direct effect on residual stress remains to be quantified. Layer-time control—waiting either too short (heat accumulation, sag) or too long (cold interfaces, high gradients)—emerges from BAAM thermal analyses as a schedulable mitigation variable [66,87]. Vacuum printing and thermal–mechanical consolidation extend the same logic by changing the heat transfer and contact conditions during deposition. In vacuum-assisted PEEK printing, reduced convective cooling allows the extruded filament to remain at an elevated temperature for longer, supporting crystallization, molecular diffusion, and interlayer merging [72]. For short CF/PEEK, vacuum printing and parameter optimization have been shown to reduce porosity and improve flexural performance [73]. A complementary approach is local preheating combined with mechanical compaction. In PEEK/CF, thermodynamic coupling through preheating and impact compaction reduced porosity and improved forming quality, although excessive alteration of layer geometry may compromise interlayer properties [74]. These approaches should therefore be evaluated simultaneously by porosity, crystallinity, dimensional stability, residual strain, and interlayer strength.

### 7.2. Deposition Strategy and Design Measures

Toolpath-level measures redistribute rather than eliminate stress: alternating or concentric raster patterns lower peak stresses [47,112,147], and FEM-driven parameter optimization (Taguchi/ANOVA on speed, layer thickness, temperatures) yields warpage reductions without hardware changes [40,88,138]. Design measures follow from the geometry effects of Section 3.4: rounded corners and circular sections outperform sharp-cornered equivalents [34], large flat bases should be avoided or anchored with brims/rafts, and platform adhesion engineering prevents premature stress release during the build [37]. In-process monitoring with camera-based neural networks closes the loop by detecting warp onset and correcting parameters before failure [155,156,157].

### 7.3. Feedstock Measures

Reducing the eigenstrain at the source is the most robust mitigation. Low CTE fillers (short CF, glass spheres, perlite, CNT) directly cut the driving force [7,15,75,81], matrix selection or blending toward lower crystallization shrinkage tames semi-crystalline systems [68], and slow-crystallizing PAEK grades show that polymer architecture can widen the processing window while reducing stress [68]. The trade-offs are increased porosity and melt viscosity and possibly reduced ductility [24,31].

### 7.4. Post-Process Annealing

Annealing above Tg relaxes locked-in stress and, for semi-crystalline polymers, raises crystallinity. For PLA, annealing increases crystallinity and flexural performance by 11–17% [89,166]. For ABS, annealing below Tg stiffens but embrittles, while annealing at or above Tg restores ductility [52,169]. PEEK is the strongest case: heat treatment lifts crystallinity from ~16% to ~29% with corresponding strength gains, compensating for cold-chamber printing [32,167], with treatment temperature and infill jointly determining the outcome [168] and near-melt annealing additionally welding layers [53]. Two-step protocols optimize crystal populations in PAEKs [68]. The principal risks are dimensional change and creep during treatment—shrinkages of several percent are reported, mitigated by fillers (recycled CF halves annealing shrinkage of ABS) and by fixturing [160,169]. Annealing therefore exchanges residual stress for a controlled, anticipated distortion, which can be pre-compensated in CAD [169].

### 7.5. Trade-Off Synthesis

No single measure is dominant: thermal management reduces stress but increases energy cost and may slow crystallization-dependent strength development, fillers reduce shrinkage but raise porosity, annealing relieves stress but distorts geometry, and stiff infills reduce distortion while storing more stress [83]. Rational practice combines feedstock-level CTE reduction, chamber/local heating sized to the polymer’s stress-freezing window, toolpath optimization, and, where tolerances permit, post-annealing with dimensional pre-compensation. A critical distinction is required between residual-stress mitigation demonstrated by direct stress or strain measurement and mitigation inferred from indirect indicators such as reduced warpage, lower porosity, higher crystallinity, or improved mechanical strength. Many thermal and vacuum-based strategies provide convincing indirect evidence, but their quantitative effect on the residual-stress field remains insufficiently validated.

### 7.6. Industrial Implications of Residual-Stress Mitigation

From an industrial perspective, residual-stress mitigation must be evaluated not only by its ability to reduce warpage, but also by its effect on productivity, cost, dimensional repeatability, part qualification, and process robustness. Heated chambers and controlled build platforms are effective for high-temperature polymers such as PEEK, PEKK, PPS, and PEI, but they increase equipment cost, energy demand, thermal stabilization time, and safety requirements [32,38,136]. Local laser, infrared, or auxiliary preheating can reduce thermal gradients and improve interlayer bonding, but its industrial use requires controlled temperature management to avoid local overheating, polymer degradation, or excessive crystallization [69,70,71]. Vacuum-assisted printing is promising for PEEK and PAEK composites because it reduces convective heat loss and can improve consolidation, but it increases machine complexity and currently remains supported mainly by indirect residual-stress evidence [72,73].

Toolpath optimization and design adaptation are more easily transferable to production because they require limited hardware modification. Alternating, concentric, or geometry-adapted raster strategies can reduce stress concentrations and improve dimensional repeatability, but they may also change mechanical anisotropy and load-path efficiency [47,112,147]. Therefore, toolpath changes must be assessed together with the functional load case of the part, not only with warpage metrics. Feedstock-level strategies, including low-CTE fillers, short fibers, and slow-crystallizing matrices, are attractive because they reduce the shrinkage driving force at the material level [7,15,75,81]. However, they may increase melt viscosity, nozzle wear, porosity, brittleness, material cost, and batch-to-batch variability. In continuous-fiber systems, residual-stress control also becomes part of fiber-path design, because fiber orientation, curvature, local tension, and impregnation quality affect both stress retention and structural performance.

**Figure 7:**
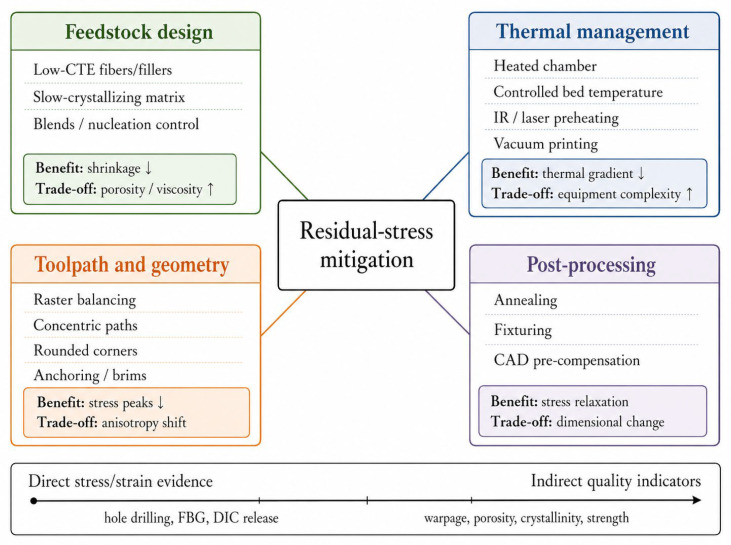
Parameter–mechanism–response map for residual-stress mitigation. The symbols ↑ and ↓ indicate an increase and a decrease. The bottom horizontal arrow represents a continuum from direct stress/strain evidence to indirect quality indicators.

Post-process annealing is industrially relevant when stress relaxation and crystallinity development are required, but it converts residual-stress mitigation into a dimensional-control problem [52,53,89,166,167,168,169]. Annealing may improve strength, ductility, or interlayer welding, but it can also cause additional shrinkage, creep, or loss of tolerance. For production parts, annealing should therefore be coupled with fixtures, dimensional pre-compensation in CAD, and post-process inspection. Overall, the industrial implementation of residual-stress mitigation requires a combined process window that balances residual stress, warpage, porosity, crystallinity, interlayer strength, cycle time, and cost. Strategies supported only by reduced warpage should be treated as preliminary until confirmed by residual-strain, residual-stress, or validated model-based evidence, particularly because lower visible distortion can coexist with higher retained stress in reinforced systems [83]. The parameter–mechanism–response relationships are summarized in Figure 7.

## 8. Challenges and Future Directions

Standardization—There is no dedicated standard for residual-stress measurement in printed polymers or printed composites. Isotropy assumptions are invalid for the orthotropic, porous FFF mesostructure [30,117]. Round-robin campaigns and an adaptation of hole-drilling/slitting procedures with FEA-based calibration for printed laminates are needed [114,115].

Direct versus indirect evidence—Future studies should explicitly distinguish direct residual-stress evidence from indirect process quality indicators. Hole drilling, layer removal, slitting, curvature reconstruction, embedded FBG sensing, DIC-based release measurements, and photoelastic stress relief methods provide direct or semi-direct information on stress or released strain. In contrast, warpage, porosity, crystallinity, interlayer strength, and mechanical performance are indirect indicators that require model-based interpretation. This distinction is particularly important for reinforced feedstocks, where reduced visible distortion can coexist with higher stress retained in a stiffened structure.

Continuous-fiber and high-temperature PAEK parts—Quantitative residual-stress data for continuous-fiber FFF are absent despite the technology’s structural ambitions. Recent studies on continuous CF/PEEK show that vacuum printing and infrared preheating improve consolidation, interfacial bonding, and mechanical performance [158,159], but they do not yet provide spatially resolved residual-stress fields. Systematic studies should link fiber path, fiber volume fraction, fiber tension, nozzle temperature, chamber temperature, vacuum level, infrared preheating, porosity, crystallinity, residual strain, and post-release distortion. Embedded FBG sensors, full-field DIC, reflection photoelasticity, micro-CT, DSC, and thermo-mechanical simulation provide a realistic combined toolset for this task.

AI-driven optimization and real-time residual-stress detection—A major future direction is the development of AI-assisted residual-stress control that combines physics-based simulation, reduced-order modeling, process monitoring, and experimental calibration [48,49,51,111]. Machine learning models should not be trained only on warpage or dimensional-error data, because these outputs do not uniquely represent the retained stress state in reinforced composites. More reliable workflows should integrate validated thermo-mechanical simulations with in situ temperature fields, deflection measurements, embedded strain sensors, and optical monitoring [124,125,154,155,156,157].

Real-time residual-stress detection remains more challenging than real-time warpage detection because residual stress is not directly observable from surface shape alone. Future monitoring systems should therefore treat residual stress as a hidden process state estimated from multiple indirect signals, including thermal history, local cooling rate, strain evolution, substrate deflection, crystallization state, and defect formation. Embedded FBG sensors, DIC, in situ deflection monitoring, infrared thermography, and photoelastic or other optical stress indicators could provide complementary data streams for model updating and digital-twin frameworks [50,84,124,125,129]. Viscoelastic relaxation and aging—The post-print evolution of residual stress, including viscoelastic relaxation, physical aging, and environmental or hygroscopic effects in natural-fiber composites [76], is rarely quantified, although it is important for the dimensional stability of functional parts [170].

Sustainability—Recycled and bio-based fiber feedstocks exhibit different shrinkage and stress behavior [24,160,162]; their residual-stress characterization should accompany their mechanical qualification.

Designed residual stress—4D printing demonstrates that process-induced stresses can be programmed [45,46,47]. The same toolset could deliberately introduce beneficial compressive surface stresses (printed prestressing), an essentially unexplored concept for which the strength correlation reported in [42] provides initial evidence.

## 9. Conclusions

This review synthesized residual-stress formation, measurement, prediction, and mitigation in reinforced thermoplastic composites produced by material extrusion additive manufacturing. The main scientific conclusion is that residual stress in FFF/MEX is a coupled multiscale phenomenon governed by constrained thermal contraction, crystallization shrinkage, reinforcement-induced anisotropy, fiber–matrix thermal expansion mismatch, porosity, and process-imposed boundary conditions. These mechanisms are strongly dependent on the matrix type, reinforcement architecture, print path, mesostructure, and thermal history.

A central technical finding is that reduced distortion is not equivalent to reduced residual stress. Fibers, fillers, and nanofillers can reduce the effective coefficient of thermal expansion and suppress visible warpage, curling, and dimensional deviation. At the same time, reinforcement increases stiffness, introduces anisotropic constraint, modifies crystallization behavior, and may increase porosity. Reinforced parts can therefore retain substantial internal residual stress even when macroscopic distortion is small. Warpage should consequently be treated as an indirect deformation metric rather than as a sufficient residual-stress metric.

A major contribution of this review is the classification and critical comparison of measurement methods according to evidence type, measurement mode, and dominant assessment scale. Relaxation-based methods such as hole drilling, layer removal, slitting, and curvature reconstruction provide the most direct residual-stress or released-strain information, but require material-specific calibration for orthotropic, porous, and viscoelastic printed composites. Embedded FBG sensors, DIC, reflection photoelasticity, thermography, micro-CT, DSC, mechanical testing, and warpage metrology provide complementary information, but most of these techniques require model-based interpretation. The most reliable assessment is therefore a combined workflow linking residual-stress measurement, full-field deformation, thermal history, crystallinity, porosity, and fiber orientation.

From the prediction perspective, the field has progressed from analytical layerwise shrinkage models toward sequential thermo-mechanical finite-element simulations, crystallization-coupled thermo-viscoelastic models, fiber-orientation-aware simulations, and reduced-order or data-driven approaches. However, many models remain validated mainly by temperature fields, warpage, or global distortion. Direct residual-strain or residual-stress validation remains limited, particularly for continuous-fiber FFF parts and high-temperature PAEK-based composites. This limits the quantitative reliability of current stress-field predictions.

Residual-stress mitigation is most effective when several strategies are combined. Feedstock-level measures reduce shrinkage through low-CTE fibers, fillers, blends, or slow-crystallizing matrices. In-process thermal management by heated chambers, controlled bed temperature, local laser or infrared preheating, vacuum printing, and thermo-mechanical consolidation reduces thermal gradients and improves interlayer bonding. Toolpath and geometry optimization redistribute stress, while post-process annealing relaxes frozen-in stress but introduces dimensional change. No individual strategy is universally optimal because each route involves trade-offs in porosity, crystallinity, stiffness, interlayer bonding, dimensional accuracy, energy demand, and process complexity.

Overall, the main merit of this review is to reframe residual stress in reinforced material extrusion as a quantitative stress-management problem rather than a qualitative warpage-reduction problem. Future progress requires standardized measurement protocols for porous and orthotropic printed composites, spatially resolved residual-stress data for continuous-fiber and high-temperature systems, validated coupled thermo-mechanical models, and closed-loop process control capable of either suppressing undesirable residual stress or deliberately programming beneficial states.

## Figures and Tables

**Table 1:** Classification of traditional and advanced reinforced thermoplastic composites used in material extrusion additive manufacturing.

Class	Typical Material Systems	Reinforcement Architecture	Relevance to Residual Stress and Distortion	References
Traditional particle-filled thermoplastics	Mineral-filled, glass-sphere-filled, wood-filled, metal-powder-filled thermoplastics	Discrete particles dispersed in the polymer matrix	Modify CTE, shrinkage, crystallization, viscosity, and dimensional stability; residual-stress evidence is usually indirect through warpage or shrinkage	[75,76,81]
Traditional nanofilled thermoplastics	CNT-, graphene-, or carbon-nanofiller-modified ABS/PLA systems	Nanoscale dispersed reinforcement or conductive networks	Can reduce shrinkage and improve thermal-dimensional stability, but dispersion quality and viscosity affect voids and bonding	[7,8,82]
Traditional short-fiber composites	Short carbon- or glass-fiber-reinforced ABS, PLA, PA, PP	Discontinuous fibers aligned mainly by melt flow and print path	Reduce visible warpage through lower effective CTE and higher stiffness, but may increase retained residual stress in a stiffer structure	[9,10,11,24,31,83]
Advanced continuous-fiber composites	Continuous carbon-, glass-, or aramid-fiber-reinforced PLA, PA, PEEK, or related matrices	Continuous fibers deposited along prescribed toolpaths	Strong path-dependent stiffness, anisotropic shrinkage, fiber tension, interfacial bonding, and orientation-dependent residual strain	[12,13,14,21,22,23,77,78,79,80,84]
Advanced high-temperature composites	PEEK, PEKK, PAEK, PPS and their carbon-fiber-reinforced variants	Short or continuous reinforcement in high-temperature semi-crystalline matrices	Strong coupling of thermal contraction, crystallization shrinkage, chamber temperature, interlayer bonding, and residual stress	[32,33,38,39,68,85,86]
Advanced large-format reinforced feedstocks	BAAM-grade CF/ABS, CF/PETG, CF/PPS-type systems	High filler or short-fiber content in large deposited beads	Reduced macroscopic distortion enables large-scale printing, but thermal gradients, bead size, anisotropic CTE, and slow cooling require process-level stress control	[15,16,17,66,67,87]

**Table 2:** Representative application classes of reinforced thermoplastic material extrusion and associated residual-stress and distortion challenges.

Application Class	Representative Material Systems	Main Functional Requirement	Residual-Stress and Distortion Relevance	References
Functional and adaptive components	CNT- or graphene-modified ABS/PLA; wood-fiber and wood–plastic composites	Electrical or thermal functionality, dimensional stability, and stimulus-responsive deformation	Fillers modify thermal expansion, shrinkage, heat transfer, viscosity, and void formation. In adaptive structures, process-induced or hygroscopic strain may be deliberately retained to generate shape change. Direct residual-stress measurements remain limited.	[7,8,31,45,46,47,76,82]
Large-format tooling and demonstrator structures	Short-CF/ABS and related BAAM-grade carbon-fiber-reinforced thermoplastics	Meter-scale deposition, dimensional stability, tooling, and large structural demonstrators	Carbon fibers reduce effective thermal expansion and visible distortion, enabling large-scale printing. However, large bead dimensions, slow cooling, anisotropic properties, layer time, and platform release generate part-scale thermal gradients and distortion.	[15,16,17,66,67,87]
Lightweight load-bearing structural components	Continuous carbon-, glass-, or aramid-fiber-reinforced PLA, PA, PEEK, and related thermoplastics	High directional stiffness and strength with fiber paths aligned to the principal loading direction	The deposited fiber trajectory controls local stiffness, contraction constraint, fiber tension, void formation, impregnation quality, and interfacial bonding. Residual strain is therefore strongly dependent on fiber orientation, lay-up, and toolpath curvature.	[12,13,14,21,22,23,77,78,79,80,84]
High-temperature engineering and biomedical components	PEEK, PEKK, PAEK, PPS, and their short- or continuous-fiber-reinforced variants	Thermal and chemical resistance, structural performance, dimensional accuracy, and biomedical applicability	High processing temperatures and semi-crystalline matrices produce strong coupling between thermal contraction, crystallization shrinkage, porosity, interlayer bonding, and chamber conditions. Insufficient thermal control may cause warpage, delamination, and cracking.	[32,33,38,39,68,72,73,74,85,86]
Programmed 4D and morphing structures	PLA, ABS, and wood–plastic composites with tailored raster architectures	Controlled thermally or environmentally activated shape change	Residual strain is intentionally programmed through anisotropic raster paths, differential contraction, and material response. In these systems, residual stress is a functional design variable rather than only an undesirable manufacturing defect.	[45,46,47,76]

**Table 3:** Matrix-dependent residual-stress mechanisms and control priorities in material extrusion additive manufacturing.

Matrix Class and Representative Polymers	Dominant Residual-Stress Mechanism	Critical Process Variables	Characteristic Manifestation and Control Priority	References
Amorphous thermoplastics: ABS, ASA, PC, PETG, and PEI	Constrained thermal contraction as molecular mobility decreases below the glass-transition or stress-freezing range	Chamber and ambient temperature, cooling rate, temperature interval below the stress-freezing temperature, and time available for viscoelastic relaxation	Warpage, corner lift, and dimensional deviation. The primary control priority is reduction in thermal gradients and extension of the relaxation window during deposition.	[29,30,35,88]
Moderately crystallizing semi-crystalline thermoplastics: PLA and related matrices	Coupled thermal contraction and crystallization-induced volumetric shrinkage	Cooling rate, local reheating, nucleation behavior, crystallization kinetics, and post-process annealing history	Warpage and spatially non-uniform crystallinity. Control requires a defined thermal history and, where applicable, controlled annealing with allowance for dimensional change.	[89,90]
High-shrinkage semi-crystalline thermoplastics: PP and filled PP systems	Pronounced crystallization shrinkage combined with limited build-platform adhesion	Chamber and bed temperature, cooling conditions, platform adhesion, filler type, and filler content	Severe warpage, corner lift, and premature platform detachment. The main priorities are chamber-temperature control, adhesion optimization, and shrinkage-reducing fillers.	[37,75,81,93]
High-temperature semi-crystalline matrices: PEEK, PEKK, PAEK, PPS, and their reinforced variants	Large thermal contraction interval coupled with crystallization shrinkage, temperature-dependent relaxation, and incomplete interlayer bonding	Chamber temperature, extrusion temperature, local preheating, vacuum conditions, cooling rate, crystallization kinetics, and post-process thermal treatment	Warpage, delamination, cracking, porosity, and dimensional instability. Effective control requires high-temperature thermal management, extended bonding time, and, where suitable, slow-crystallizing matrix grades.	[32,33,38,39,68,72,73,74,85,86,92]

**Table 4:** Comparison of residual-stress measurement and characterization methods for FDM/FFF parts and printed composites.

Method	Principle	Destructiveness	In Situ Capable	Output	Suitability for Printed Composites	References
Incremental hole drilling (strain gauge/ESPI/DIC)	Strain relief around drilled hole	Semi-destructive	No	In-depth stress profile (near-surface)	Requires orthotropic FEA calibration; porosity biases results; ESPI/DIC avoid gauge bonding on compliant surfaces	[30,113,114,115,116,117]
Layer removal/sectioning (+DIC)	Deformation after material removal	Destructive	No	Layerwise stress/released strain field	Natural fit to layered FFF builds; established for laminates	[31,118,119]
Crack compliance/slitting	Strain released by incremental slot	Destructive	No	Through-thickness profile	Validated for layered composites; FFF-specific use scarce (gap)	[120,121,122]
Curvature/bridge methods (+optical scan, FEM inversion)	Distortion of released or asymmetric specimens	Destructive (release)	Partly	Average stress/stress state via model	Simple; couples directly to strength assessment	[29,42]
Embedded FBG sensors	Bragg wavelength shift from strain	Non-destructive (embedded)	Yes	Local strain history during build + residual strain	Demonstrated in ABS, PLA, PA and continuous-fiber parts; perturbs mesostructure locally	[85,105,106,123]
Digital image correlation	Full-field surface displacement	Non-destructive	Yes	Distortion/released strain fields	Uses natural bead texture; model inversion needed for stress	[31,124,125]
In situ deflection monitoring	Substrate/cantilever deflection during build	Non-destructive	Yes	Scalar stress-buildup proxy	Simple validation channel for process FEA	[48]
X-ray diffraction	Lattice strain of crystalline phases	Non-destructive	No	Crystal-phase stress	Limited to semi-crystalline fraction/crystalline fillers	[42]
Stress whitening	Stress-induced optical whitening	Non-destructive	No	Qualitative stress indicator	Demonstrated for transparent injection-molded polymers; applicability to FFF requires validation	[126]
Photoelasticity (+HDM)	Stress birefringence	Non-destructive	No	Qualitative full-field map (quantitative with HDM)	Transparent amorphous polymers only (e.g., PLA)	[50,127]
IR thermography	Surface temperature field	Non-destructive	Yes	Thermal history (model input/validation)	Indirect—supports all model-based methods	[26,66,87]
Warpage metrology (corner lift, scanning, LVDT)	Integrated distortion of part	Non-destructive	Partly	Distortion metrics	Universal comparator, but confounds stress with stiffness/constraint	[35,36,37,107]

**Table 5:** Representative residual-stress/distortion simulation studies for material extrusion AM.

Study	Material	Approach/Tool	Coupled Physics	Validation Level	Key Finding
Wang et al., 2007 [29]	ABS	Analytical (layerwise contraction)	Thermal shrinkage	Warpage experiments	Closed-form warp vs. layers/section/ΔT
Armillotta et al., 2018 [35]	ABS	Analytical + DOE	Thermal shrinkage	Warpage experiments	Warpage scaling with part dimensions
Zhang & Chou 2006/2008 [132,133]	ABS	FEA, element activation (ANSYS)	Thermo-mechanical	Qualitative	Framework; speed/layer/road parametrics
Cattenone et al., 2019 [111]	ABS	FEA (Abaqus), birth-death	Thermo-mechanical	Distortion measurements	Mesh/time-step/material-model sensitivity
El Moumen et al., 2019 [134]	Polymer composites	FEA	Thermo-mechanical	Literature data	Temperature + stress fields in reinforced parts
Samy et al., 2021–2022 [93,112,135,136]	PP, ABS	COMSOL Multiphysics, element activation	+Crystallization kinetics, Maxwell viscoelasticity	Distortion experiments	Ambient T, speed, raster effects; amorphous vs. semi-crystalline
Brenken/Barocio (Purdue) 2017–2022 [33,85,86,109]	50 wt.% CF/PPS	Abaqus UMAT/UMATHT (Additive3D)	+Crystallization, thermoviscoelasticity, fusion bonding	EDAM prints	Validated composite EDAM simulation chain
Talagani et al., 2015 [16]	CF/ABS (BAAM)	GENOA multiscale FEA	Thermo-mechanical, fiber orientation	Full-size printed car	First structure-scale composite AM simulation
Kim et al., 2019 [67]	20 wt.% CF/ABS (BAAM)	De-homogenization + FEA	+Anisotropic CTE (TMA), fiber orientation	IR + LVDT on 4-ft wall	Orientation-resolved distortion prediction
Trofimov et al., 2022 [137]	PLA/composite	FEA, optimized activation	Thermo-mechanical	Temperature + distortion	Validated, cost-reduced workflow
Jiang et al., 2023 [34]	SCF/PLA	Multi-physics FEA	+Crystallinity	DSC, warpage, geometry	Corner stress concentration; geometry effect

**Table 6:** Influence of process and material variables on residual stress and warpage in FFF (↑ increase, ↓ decrease, ↓↓ a pronounced decrease, → a change from the initial value to the final value, and ~ weak/non-monotonic).

Variable (Increase)	Residual Stress	Warpage/Distortion	Mechanism	References
Chamber/ambient temperature	↓	↓	Smaller ΔT below stress-freezing point; slower cooling	[32,75,136]
Bed temperature/adhesion	~	↓ (prevents detachment)	First-layer constraint and anchoring	[37,88]
Local pre-deposition heating (IR/laser/auxiliary)	↓	↓↓ (12.3→~1 mm reported)	Interface reheating, gradient reduction	[69,70,71]
Nozzle temperature	~	~	Better bonding vs. larger contracting interval	[88,101]
Printing speed	↑	↑	Shorter reheating window, faster cooling, more porosity	[31,135]
Layer thickness	↓	↓	Fewer thermal cycles, higher thermal mass	[133,146]
Infill density	↑ (locked-in)	↓	Stiffer structure stores stress, resists distortion	[83]
Raster 0° → alternating/concentric	redistributed ↓ peaks	↓	Balanced contraction directions	[47,112,147]
Part in-plane size	↑	↑	Longer constrained shrinkage paths	[29,35]
Sharp corners (vs. rounded)	↑ local	↑	Geometric stress concentration	[34,48]
Short-fiber/filler content	↑ (in stiffened part)	↓↓	CTE reduction + stiffening; porosity ↑	[15,31,81,83]
Vacuum	↓ (indirectly inferred)	↓↓	Slower cooling, reduced convective heat loss, longer bonding and crystallization window	[72,73]

**Table 7:** Quantitative comparison of reinforcement effects relevant to residual stress and distortion in material-extruded thermoplastic composites.

Reinforcement	Content	Reported Quantitative Effect	Residual-Stress Interpretation	References
CNT or carbon nanofillers/ABS	Nanofilled filament	Shrinkage and residual deformation lower than neat ABS; improved thermal-dimensional stability reported.	Lower shrinkage; stress inferred indirectly.	[7,31,82]
Short CF/ABS, BAAM CF/ABS	13–20 wt.% CF	Distortion sufficiently reduced for “out-of-oven” large-format printing.	Warpage decreases through CTE reduction and stiffening.	[9,15,17,66]
Short CF/PLA	SCF/PLA sections	Square sections: local stress up to ~108 MPa; circular sections: lower and more uniform stress.	Geometry and fiber-induced anisotropy control stress concentration.	[34]
Glass fiber/ABS	Increasing fiber ratio and infill	Warpage decreases, but thermal residual stress increases.	Reduced distortion may mask higher locked-in stress.	[83]
Expanded perlite/PP	Perlite-filled PP	Warpage was reduced by approximately 25%.	Shrinkage-driven distortion reduced; direct stress not measured.	[81]
Glass spheres/PP	Glass-sphere-filled PP	Dimensional accuracy improved with chamber-temperature control.	Indirect evidence of shrinkage and distortion reduction.	[75]
Short CF/PEEK, vacuum printed	30 wt.% SCF; 0.5 mbar	Flexural stress and modulus increased; porosity reduced vs. atmospheric printing.	Lower porosity and slower cooling may reduce stress gradients; direct stress data lacking.	[73]
Continuous CF/PEEK	Continuous fiber; vacuum or IR preheating	Flexural performance, bonding, and microstructure improved.	Relevant to stress control, but spatial stress fields remain scarce.	[158,159]
Recycled CF/ABS or PLA	rCF-filled filament	Dimensional stability during annealing improved.	Filler-stabilized stress relaxation suggested.	[160,161,162]

**Table 8:** Effect of fiber orientation and raster architecture on residual-stress development in material-extruded reinforced thermoplastic composites.

Fiber/Raster Orientation	Dominant Anisotropic Effect	Expected Residual-Stress Effect	Distortion Implication	References
0° raster/fibers aligned with print path	High longitudinal stiffness; suppressed longitudinal CTE	Longitudinal stress concentration along deposited roads	Directional warpage; strong path dependence	[9,47,104,112,145]
90° raster	Main contraction and stiffness axis rotated transverse to reference direction	Stress components shift from longitudinal to transverse direction	Different edge-lift and curvature response than 0° raster	[47,112]
0°/90° alternating raster	More balanced in-plane stiffness and shrinkage	Peak stresses redistributed between layers	Reduced directional distortion; more complex interlayer stress state	[47,112,147]
Concentric raster	Road orientation follows part contour	Stress follows local contour; edge effects may remain critical	Often lower global warpage, but contour regions can remain critical	[47,112,147]
Short-fiber flow alignment	Fibers align with melt flow and deposition direction	Orthotropic CTE and stiffness generate inter-road and interlayer mismatch stresses	Reduced shrinkage along road; transverse shrinkage remains matrix dominated	[9,15,102,103,104]
Continuous straight fiber paths	Very high stiffness along deposited fiber trajectory	Orientation-dependent residual strain and path-dependent constraint	Low deformation along fiber path, but higher locked-in constraint is possible	[84,145,152]
Curved or steered continuous-fiber paths	Local curvature, fiber tension, and impregnation quality vary along path	Local stress concentrations may develop near turns and steering regions	Higher risk of voids, poor bonding, and local distortion	[21,22,79,80,145]

**Table 9:** Mitigation strategies for residual stress and warpage in FFF composite parts (↑ indicates an increase, ↓ a decrease, ~ an approximate value, and → a change from the initial value to the final value).

Strategy	Stage	Mechanism	Reported Outcome	Trade-Offs	References
Heated chamber/bed	In-process	Reduced thermal gradient	Mandatory for PEEK; distortion ↓ with ambient T	Energy and equipment cost	[32,38,136]
Vacuum-assisted printing	In-process	Reduced convective cooling; slower heat dissipation	PEEK crystallinity 14.9 → 27.8%; improved bonding and reduced warpage; SF-CF/PEEK flexural stress +79.75% at 0.5 mbar	Equipment complexity; evidence mostly indirect for residual stress	[72,73]
Auxiliary/local heating plate	In-process	Pre-bond interface reheating	CF/ABS warpage 12.3 → ~1 mm; strength ↑	Degradation risk >240 °C	[70]
Preheating + impact compaction	In-process	Thermal–mechanical consolidation; void closure; improved layer contact	PEEK/CF porosity 10.15 → 6.83%; warpage mechanism analyzed by FEM	May reduce interlayer properties if layer height/contact geometry is altered	[74]
IR/laser pre-deposition heating	In-process	Interface above Tg at bonding	+50% interlayer strength (laser, ABS); BAAM interlayer ↑	Hardware integration	[69,71]
Ultrasonic vibration	In-process	Enhanced chain mobility at interface	+10% adhesion; +11–17% tensile	Stress effect unquantified	[164,165]
Toolpath/raster optimization	In-process	Balanced contraction directions	Lower peak stresses (concentric/alternating)	Possible strength anisotropy shift	[47,112,147]
Parameter optimization (DOE/FEM)	In-process	Combined thermal/kinematic tuning	Warpage minimized without hardware change	Material-specific recipes	[40,88,138]
ML-based in-process monitoring	In-process	Early warp detection + correction	>99% detection accuracy; closed-loop correction	Training data needs	[155,156,157]
Design: rounded sections, anchoring	Design	Avoid geometric concentration	Circular sections ~uniform stress	Geometric freedom reduced	[34,37]
Low-CTE fillers (CF, glass, perlite, CNT)	Feedstock	Reduced shrinkage driving force	~25% PP warpage ↓ (perlite); “out-of-oven” BAAM	Porosity ↑, ductility ↓	[7,15,31,81]
Matrix selection (slow-crystallizing PAEK, blends)	Feedstock	Reduced/retarded crystallization shrinkage	PEKK achieves approximately 80% of injection-molded strength in the as-printed state, compared with approximately 25% for PEEK.	Material cost/availability	[68]
Annealing above Tg	Post-process	Stress relaxation + crystallinity ↑	PLA flexural +11–17%; PEEK crystallinity 16 → 29%	Dimensional change (shrinkage %)	[52,166,167,168]

## Data Availability

No new data were created or analyzed in this study. Data sharing is not applicable to this article.

## Abbreviations

The following abbreviations are used in this manuscript:

AM	Additive manufacturing
MEX	Material extrusion
FDM	Fused deposition modeling
FFF	Fused filament fabrication
BAAM	Big Area Additive Manufacturing
ABS	Acrylonitrile butadiene styrene
ASA	Acrylonitrile styrene acrylate
CAD	Computer-aided design
CF	Carbon fiber
CFRP	Carbon-fiber-reinforced polymer
CNT	Carbon nanotube
CTE	Coefficient of thermal expansion
DIC	Digital image correlation
DOE	Design of experiments
DSC	Differential scanning calorimetry
EDAM	Extrusion deposition additive manufacturing
ESPI	Electronic speckle pattern interferometry
FBG	Fiber Bragg grating
FEA	Finite element analysis
FEM	Finite element method
HDM	Hole-drilling method
IR	Infrared
LVDT	Linear variable differential transformer
ML	Machine learning
ORNL	Oak Ridge National Laboratory
PA	Polyamide
PA6	Polyamide 6
PAEK	Polyaryletherketone
PC	Polycarbonate
PEEK	Polyether ether ketone
PEI	Polyetherimide
PEKK	Polyetherketoneketone
PETG	Polyethylene terephthalate glycol
PLA	Poly(lactic acid)
PP	Polypropylene
PPS	Polyphenylene sulfide
SCF	Short carbon fiber
Tg	Glass transition temperature
TMA	Thermomechanical analysis
UMAT	User material subroutine
UMATHT	User material heat transfer subroutine
WPC	Wood-plastic composite
AI	Artificial intelligence
ANOVA	Analysis of variance
CT	Computed tomography
rCF	Recycled carbon fiber
